# Micronutrients, Phytochemicals and Mediterranean Diet: A Potential Protective Role against COVID-19 through Modulation of PAF Actions and Metabolism

**DOI:** 10.3390/nu13020462

**Published:** 2021-01-30

**Authors:** Paraskevi Detopoulou, Constantinos A. Demopoulos, Smaragdi Antonopoulou

**Affiliations:** 1 Department of Clinical Nutrition, General Hospital Korgialenio Benakio, 11526 Athens, Greece; viviandeto@gmail.com; 2 Laboratory of Biochemistry, Faculty of Chemistry, National & Kapodistrian University of Athens, 16121 Athens, Greece; demopoulos@chem.uoa.gr; 3 Laboratory of Biology, Biochemistry and Microbiology, Department of Nutrition and Dietetics, School of Health Science and Education, Harokopio University, 70 El. Venizelou Street, 17671 Athens, Greece

**Keywords:** platelet activating factor, thrombosis, inflammation, Mediterranean diet, PAF-inhibitors

## Abstract

The new coronavirus disease 2019 (COVID-19) pandemic is an emerging situation with high rates of morbidity and mortality, in the pathophysiology of which inflammation and thrombosis are implicated. The disease is directly connected to the nutritional status of patients and a well-balanced diet is recommended by official sources. Recently, the role of platelet activating factor (PAF) was suggested in the pathogenesis of COVID-19. In the present review several micronutrients (vitamin A, vitamin C, vitamin E, vitamin D, selenium, omega-3 fatty acids, and minerals), phytochemicals and Mediterranean diet compounds with potential anti-COVID activity are presented. We further underline that the well-known anti-inflammatory and anti-thrombotic actions of the investigated nutrients and/or holistic dietary schemes, such as the Mediterranean diet, are also mediated through PAF. In conclusion, there is no single food to prevent coronavirus Although the relationship between PAF and COVID-19 is not robust, a healthy diet containing PAF inhibitors may target both inflammation and thrombosis and prevent the deleterious effects of COVID-19. The next step is the experimental confirmation or not of the PAF-COVID-19 hypothesis.

## 1. Introduction

The new coronavirus disease 2019 (COVID-19) pandemic is an emerging situation with high rates of infectivity, morbidity and mortality [1]. The pathophysiology of the disease involves a cytokine storm and the activation of thrombotic pathways [2]. It was recently documented in Wuhan, China, that the disease is directly connected to the nutritional status of severely and critically ill patients [3]. Although “there is no diet to prevent coronavirus” [4] and there are limited applied clinical nutrition protocols for COVID-19 patients [5,6,7], the focus of the international community shifts to recommending a healthy dietary pattern [8], intended to control inflammation and thrombosis, which accompany the syndromes’ complications [2]. Indeed, a well-balanced diet ensures the proper functioning of the immune system [4] and several micro-constituents alone or as part of a healthy dietary pattern, such as the Mediterranean diet, play a role in viral infections [9], inflammation [10] and thrombosis [11,12]. A key molecule implicated in COVID-19 pathology is platelet activating factor (PAF), as recently highlighted by our group [2,13]. More particularly, PAF is a glyceryl-ether phospholipid (1-O-alkyl-2-acetyl-*sn*-glycero-3-phosphocholine) [14], which is a potent mediator of inflammation and thrombosis [15,16]. It is produced by various cells such as platelets, endothelial cells, macrophages, monocytes, neutrophils and other cells continuously or upon inflammatory stimuli [15]. It is noted that the main biosynthetic enzymes of PAF are lyso-PAF-acetyltransferases and dithiothreitol-insensitive CDP-choline: 1-alkyl-2-acetyl-*sn*-glycerol cholinephosphotransferase (PAF-CPT). PAF is catabolized by PAF acetylhydrolase or lipoprotein associated phospholipase A_2_ (Lp-PLA_2_) [15]. PAF levels, PAF induced platelet aggregation and the activity of its metabolic enzymes correlate with various clinical states such as asthma, stroke, atherosclerosis, heart failure, cancer, kidney disease and viral diseases [9,15,17].

With respect to COVID-19, PAF is a highly pyrogenic agent [18] and it affects the activity of angiotensin converting enzyme 2 (ACE2) [19], which is used as a receptor to facilitate the entrance of SARS-CoV-2 into the cells [20]. According to a lipidomic analysis human cells infected with the coronavirus HCoV-229E are enriched in PAF [21]. Moreover, oxidized phospholipids, which contain PAF and PAF-like lipids [22,23] have been detected in the respiratory system of patients with SARS-CoV-1 and seem to increase cytokine production and lung injury via Toll-like receptor (TLR)4 [24]. Another similarity between the phenotypic manifestations of COVID-19 and PAF actions is that they are both connected to Kawasaki-like disease in children [25]. PAF has been also found to increase phagocytic capacity in equine alveolar macrophages [26] and its levels are increased in acute pulmonary disease [27], pulmonary hypertension [28] and sepsis [29]. Interestingly, the first-line drugs used in the COVID-19 epidemic, such as chloroquine have been also found to reduce PAF induced pulmonary edema [30]. Hopefully, specific inhibitors such as rupatadine can modulate the action of PAF [31] and they have been proposed as potential candidate therapeutic compounds against COVID-19 [13]. Inversely, widely prescribed medicines, such as statins or antiretroviral drugs with pleiotropic actions also influence PAF [32,33].

The inhibitors of PAF found in natural products and microconstituents of the diet are of increasing interest [11,34]. In fact, diet can directly affect PAF induced platelet aggregation, PAF levels and/or the activity and expression of PAF metabolic enzymes [35] or it can act indirectly by modifying its environment (i.e., oxidative stress) [23]. Furthermore, the modulation of PAF by dietary parameters has been shown to affect the manifestation of disease [36]. Given the newly suggested role of PAF and its dietary inhibitors in relation to the COVID-19 epidemic in limited works [2,10,13,37], the scope of the present mini-review is to thoroughly present the potential anti-PAF actions of nutrients providing “protection” against COVID-19. We further suggest that the well-known anti-inflammatory and anti-thrombotic actions of micronutrients, phytochemicals and/or holistic dietary schemes are also mediated through PAF.

## 2. Micronutrients, COVID-19 and PAF

**Figure 1:**
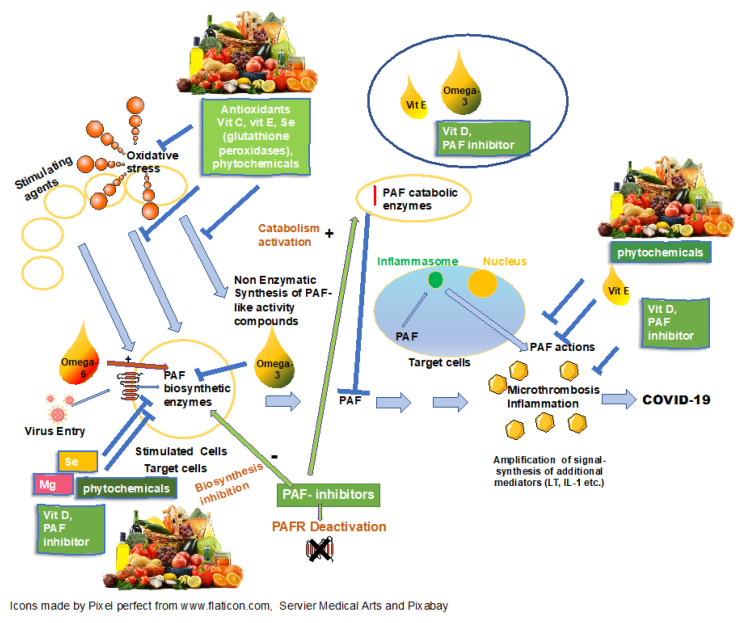
Protective role of nutrition against COVID-19 through modulation of PAF actions and metabolism. Figure legend: Various agents activate cells (usually mast cells) and secrete platelet activating factor (PAF). The produced PAF then affects various target cells (tissue-organs). In activated target cells, coronavirus 19 binds to PAF receptors (PAFRs) exposed to their pericellular membrane, enters these cells more easily and further induces PAF production. The action of PAF through inflammasomes, which is not mediated through PAFRs should not be ignored. Of course, the virus can also enter and act on the initially activated cells (usually mast cells) causing its known actions. Prolonged and replenished PAF production (feedback control) goes hand in hand with a prolonged inflammatory and prothrombotic response and the characteristic phenotypic manifestations of COVID-19. PAF inhibitors can act (i) by inactivating PAFRs and (ii) by affecting PAF metabolism. PAF inhibitors have been found to typically reduce the activity of one or both PAF key biosynthetic enzymes (regulatory enzymes) and/or increase the activity of the PAF key degrading enzyme. The role of antioxidants, micronutrients and phytochemicals that limits the initial activation by reducing oxidative stress and/or the production of PAF-like activity compounds in a non-enzymatic way is also pointed out.

Several micronutrients have been suggested to act as immunomodulatory agents against COVID-19 [38]. Their main actions along with their potential anti-thrombotic and anti-PAF effect, are briefly presented and are depicted in Figure 1.

### 2.1. Vitamin A

Carotenoids have immunoregulatory actions including reducing free radicals [39] and pro-inflammatory molecules, such as IL-2 and TNF-α. Moreover, vitamin A downregulates IFNγ production, an action which is more evident in a high oxidative stress environment [40]. Vitamin A is implicated in respiratory diseases since it plays a role in the formation of a healthy mucus layer [41] and its overt or subclinical deficiency increases morbidity and mortality from infections and respiratory diseases [41].

Retinoic acid can modulate the gene expression of PAF-receptor [42] and acts synergistically with PAF to activate the inducible prostaglandin synthase gene [43]. Prostaglandins synthesis contributes to gastric mucosal defense, although different effects are attributed to the many kinds of prostaglandins [44]. It is also noted that serum retinol has been inversely related to the activity of Lp-PLA_2_ in epidemiological studies [45]. The interplay of vitamin A and PAF in immunity is also highlighted by the fact that the host-versus- graft reaction, in which PAF is implicated [46], is enhanced by high levels of vitamin A (34).

### 2.2. Vitamin C

Vitamin C acts as an antioxidant and can boost the immune system [47]. It is involved in the function and integrity of mucosal cells, the normal functioning of T cells while it also exerts antimicrobial effects [37]. Vitamin C and concentration is high in leukocytes and it is utilized in the case of infection [48]. Vitamin C reduces the risk, the severity, and the duration of different infectious diseases, its status has been associated with pneumonia [49] and the supplementation with vitamin C may prevent and treat respiratory and systemic infections [47]. Therapeutic doses of vitamin C (24 gr/day intravenously, for seven days) are currently being tested in hospitalized COVID-19 patients [7]. However, official sources indicate that there is no evidence yet to support intravenous super doses of vitamin C in the management of COVID-19 [50].

In addition, vitamin C decreased markers of thrombosis, such as tissue plasminogen activator and von Willebrand factor in high risk patients with cardiovascular disease and diabetes [51], an action already suggested in the 1970s [52]. In the same context, it has been found to reduce PAF levels in vitro [53]. Indeed, vitamin C reduces oxidative stress [47], which is a strong trigger for synthesis of PAF [54] and its receptor [55]. It is also noted that in frailty, which worsens COVID-19 outcomes [56] the PAF catabolic enzyme Lp-PLA_2_ is increased (suggesting its upregulation to counter-balance PAF levels) while anti-oxidant status is decreased (vitamin C, E, α-tocopherol, biological anti-oxidant potential, and total thiol levels) [57]. Vitamin C status could thus affect the inflammatory and micro-thrombotic environment including PAF and the morbidity of COVID-19.

### 2.3. Vitamin D

Vitamin D exerts antimicrobial and anti-oxidant effects and supports the immune system against respiratory infection [58]. According to a meta-analysis vitamin D supplementation reduces the risk of acute respiratory infections [59], has been inversely related to hepatitis viral load [60] and improves antibacterial immunity in HIV-1 patients [61]. An inverse association between mean levels of vitamin D and the number of COVID-19 cases/1 M was recently reported in a cross-sectional European study [62] whereas a UK study did not find an association between the vitamin’s status and COVID-19 risk [63]. Moreover, low levels of vitamin D were found in COVID-19 positive patients [64] or hospitalized patients with COVID-19 [65] and have been connected to the severity of the disease. The hypothesis that vitamin D may explain susceptibility to COVID-19 infection in dark colored skin individuals does not seem to be valid [63]. It is noteworthy that several clinical trials are on the way regarding the role of vitamin D in the prevention and treatment of COVID-19, reviewed elsewhere [48].

In vitro data suggest that 1,25-dihydroxyvitamin D3 reduces the secretion of the catabolic enzyme PAF-AH from placenta macrophages [66], which implies an interrelation between PAF and the vitamin. Moreover, paricalcitol has an anti-inflammatory and anti-PAF action in hemodialysis patients inhibiting PAF/thrombin-induced platelet aggregation, reducing the activity of PAF biosynthetic enzymes and increasing the activity of the catabolic enzyme of PAF, i.e., PAF-AH [67]. The connection between vitamin D and PAF is further substantiated by the known anti-thrombotic effects of vitamin D [68].

### 2.4. Vitamin E

Vitamin E acts as an antioxidant and has a role in the proper functioning of the immune system [58]. Indeed, it protects cell membranes, including those of immune cells from lipid peroxidation [69]. In cases of influenza infection, the lung levels of vitamin E are reduced [70], and supplementation with the vitamin reduces the severity and duration of the disease [71,72]. In the same context, in a meta-analysis of randomized controlled trials vitamin E reduced C-reactive protein (CRP) levels [73]. A combination of vitamin E and C has been recently proposed for ameliorating cardiac injuries of critically ill COVID-19 patients, which furthers underline their role in the COVID-19 disease [74].

Vitamin E deficiency is connected to increased PAF synthesis in rat polynuclear cells [75]. Moreover, vitamin E inhibits PAF induced platelet aggregation [76,77,78] and PAF synthesis [79]. In addition, lycopene alone or in combination with a-tocopherol reduces PAF synthesis in stimulated endothelial cells [80], which can further blunt the inflammatory cataract. Vitamin E and increased Lp-PLA_2_ have been associated with decreased asthma development [81], and the vitamin may indirectly affect Lp-PLA_2_ since it improves LDL quality, in which the enzyme is attached [82]. However, high levels of vitamin E ingested as a supplement (1500 IU for two weeks) seem not to influence the concentration of lyso-PAF [83]. In total, vitamin E can affect PAF levels, metabolism and its actions on platelets, i.e., the pro-thrombotic state.

### 2.5. Selenium

Selenium has been proposed to potentially play a role in COVID-19 prevention, since in the form of sodium selenite it can oxidize thiol groups in the virus protein disulfide isomerase and thus inhibit the entrance viruses into the cell [84]. It also has an antioxidant role since it is a structural component of glutathione peroxidases, a family of antioxidant enzymes [85]. In parallel, selenoprotein H is involved in redox transcription while selenoprotein K found in the endoplasmic reticulum, is involved in calcium flux in immune cells which is a critical step in immune response [85]. Selenium deficiency is associated with an increase in inflammatory molecules [86], and selenium supplementation has been found to improve the response against H1N1 virus [87]. In mouse models of asthma there seems to be a reverse-U relation with selenium concentration since too little or too much contributed to asthma attenuation [88]. The relationship selenium and the immune system is further corroborated in hospitalized patients with COVID-19, in which selenium levels were found to be sub-optimal [65]. Moreover, good selenium status, as assessed by the selenium hair content, has been connected to a higher recovery rate from COVID-19 [89].

The relationship between selenium and PAF can be considered under the prism of the effects of selenium on oxidative stress and phospholipid metabolism. Firstly, the modulation of oxidative stress by selenium could affect PAF metabolism, as for example it deactivates Lp-PLA_2_ [23] and increases PAF synthesis [54]. Secondly, selenoprotein I is implicated in phospholipid biosynthesis [85]. Moreover, PAF production is increased in the case of selenium deficiency in endothelial cells [90,91] possibly through activation of its biosynthetic enzyme lyso-PAF-acetyltransferase [90]. It is noted that the content of diet in selenium did not alter Lp-PLA_2_ in rats [92], while selenium deficiency is associated with arterial thrombosis and selenium seems to decrease platelet aggregation [93]. It can be thus hypothesized that worse outcomes of COVID-19 on the grounds of selenium deficiency may be at least in part attributed to increased PAF and an associated pro-thrombotic state.

### 2.6. Omega-3 Fatty Acids

Omega-3 fatty acids have anti-inflammatory and anti-thrombotic effects [10], and they may interfere with virus entry and replication through modulation of lipid rafts [94]. The results from animal studies show that mice with Klebsiella pneumoniae or Streptococcus pneumoniae had an upregulated immune defense and less bacterial burden when fed omega-3 fatty acids [95,96]. However, it is noted that fish oil-fed mice display impaired resistance to influenza infection [97,98] denoting a more complex immunomodulating effect of omega-3 fatty acids.

Omega-3 fatty acids also exert antithrombotic effects by various mechanisms including a reduction in thromboxane synthesis [99] and PAF [100,101]. Omega-3 fatty acids are incorporated in the cell membrane and may regulate the activity of PLA_2_ and thus lyso-PAF production, which is a prodrome molecule for PAF production [102]. DHA inhibits PAF increase in cell lines [103]. Moreover, omega-3 can reduce PAF production in Human Umbilical Vein Endothelial Cells (HUVEC) [101]. In cases of endotoxemia, which is also observed in seriously ill COVID-19 patients [104], linolenic acid has been found to reduce PAF production in Sprague-Dawley rats [105]. In addition, a diet rich in fish oils (10%) has been found to reduce PAF and LTB_4_ [106]. As far as PAF enzymes are concerned, a negative association has been documented between the PAF catabolic enzyme Lp-PLA_2_ and adipose tissue omega-3 fatty acids [107] while the effects of supplementation did not change the enzyme’s activity in healthy adults [108] but decreased the enzyme in volunteers with stable angina [109] and hypertriglyceridemia [110]. It is noted that Lp-PLA_2_, increases as a result of increased PAF in order to catabolize it, thus, the trend for an inverse association of Lp-PLA_2_ with omega-3 fatty acids, implies a negative association with PAF. Moreover, results from a cross-sectional study of our group have shown that omega-6 fatty acids were positively correlated with PAF-CPT while no significant correlations were observed with omega-3 fatty acids and PAF or its enzymes [111].

### 2.7. Zinc, Copper, Magnesium and Iron

Zinc plays a role in maintaining the integrity of mucosal cells and antigen response [37]. It has antimicrobial, anti-inflammatory and antioxidant effects [37]. Moreover, it has been found to inhibit the activity and replication of coronavirus (SARS-CoV-1) [112], and it has a role in interferon-γ production [113]. Zinc deficiency can increase susceptibility to various infections, including those of the respiratory system [114]. Zinc supplementation in mechanically ventilated trauma patients was related to decreased risk of ventilator-associated pneumonia [115]. Moreover, zinc may mediate the beneficial effects of the chloroquine, a drug which is widely used against COVID-19. Indeed, chloroquine is a zinc ionophore, which increases intracellular Zn^2+^ levels [116].

Copper can prevent oxidative DNA damage and decrease inflammatory markers [37], as it is a part of antioxidant enzymes such as Zn-Cu-superoxide dismutase and ceruloplasmin [117]. Its deficiency is connected with an increased rate of infections [118], which may be related to its role in T-cell proliferation and Natural Killer (NK) activity [113]. On the other hand, macrophages can attack pathogens with high copper and as a result the concentration of copper may be found increased in lung infection [119]. However, no direct connection to COVID-19 has been identified to date.

Iron participates in the differentiation and growth of epithelial tissue and the production of reactive oxygen species, which combat pathogens [58]. Supplementary iron intake has been found to reduce respiratory infections [120] while pulmonary iron modulation represents a defensive mechanism against various respiratory pathogens [121]. Despite the important role of iron in the immune system, iron-containing enzymes are essential for the replication of coronavirus [122] and the chelation of iron compounds may prove beneficial [37]. Iron can also modulate interferon production [113].

The interrelation between PAF and zinc, copper and magnesium is not very clear. A low zinc diet reduces platelet aggregation suggesting a role of this nutrient in hemostasis [123], while zinc and copper chelate complexes have a PAF inhibitory activity mainly attributed to stereochemical interactions [124,125]. Chelating agents such as Mg^2+^, reduce the activity of PAF biosynthetic enzymes, such as Lyso-PAF-acetyltransferase [126]. The relationship between PAF and copper and iron with has been investigated under the prism of copper and iron induced oxidation of lipids and PAF-related enzymes [23]. It is noted that metal- induced oxidative stress in the presence of superoxide can inactivate PAF acetylhydrolase [127] and thus potentially increase PAF levels. In addition, macrophage responsiveness to PAF is altered by interferon [128] and provides protection against PAF induced injury [129], which may reflect an indirect connection of some minerals with PAF through interferon.

### 2.8. Phytochemicals

Phytochemicals, such as polyphenols, act as antioxidants, modulate LDL oxidation [130], and also exert anti-inflammatory, antiplatelet [131] and antiviral activity [132]. Resveratrol, is an inhibitor of SARS-CoV-1 [133] and curcumin was recently reported to bind to the target receptors of SARS-CoV-2 [134]. In addition, curcumin combined with vitamin C glycyrrhizic acid promotes interferons production and has immunomodulatory properties [135]. Luteolin binds to the surface spike protein of SARS-Cov-2 inhibiting in this way its entry into cells and it is a potential inhibitor of SARS-CoV-2’s main protease (SARS-CoV 3CL) [136]. Moreover, lignans exhibit antiviral activity [137].

With respect to PAF, resveratrol and tyrosol as well as their acetylated derivatives inhibit PAF induced platelet aggregation [138] while curcumin is a PAF inhibitor [139] and plays a role in thrombosis and coagulation [140,141]. Moreover, curcumin and phenolic compounds acting as antioxidants can modulate LDL oxidation [130] and the subsequent production of PAF and PAF-like lipids [22]. Their effect could be also directly exerted on PAF biosynthetic enzymes as demonstrated by in vitro studies. Indeed, resveratrol and quercetin can inhibit both PAF’s main biosynthetic enzymes in vitro [142,143], phenolic compounds reduce the activity of PAF biosynthetic enzymes in cell cultures stimulated with IL-1β [144] and flavonoids, have been documented to reduce lyso-PAF acetyltransferase activity [145]. More particularly, pro-anthocyanidins [146], luteolin [145], quercetin [80,147], hesperidin [147] and naringin [147] reduce the activity of lyso-PAF acetyltransferase in cell lines. Licoricidin and other components were also documented to inhibit lyso-PAF acetyltransferase [148]. Moreover, the antioxidant capacity of the diet, which is at least in part affected by phytochemical intake, was inversely related with PAF levels and the activity of lyso-PAF-acetyltransferase in healthy volunteers as evidenced by our group [34]. In parallel, PAF has been inversely related to antioxidant-rich foods (herbal drinks and coffee) [34]. Lignans, which have been proposed as an anti-COVID compound [137] are also PAF inhibitors [149]. Thus, the effects of flavonoids and other phytochemicals on PAF levels, actions, and its metabolic enzymes generate the hypothesis that their anti-inflammatory and anti-thrombotic actions are at least in part mediated by the PAF circuit.

Interestingly, certain natural flavonoids also have anti-PAF activity, in addition to their anti-inflammatory actions and ability to block coronavirus from binding to target cells [2,13,136].

## 3. Mediterranean Diet, Mediterranean Foods, COVID-19 and PAF

The Mediterranean diet including olive oil, fish, honey, fruits, vegetables and herbs is rich in polyphenols and other micro-constituents [35] and it has been inversely related to respiratory diseases [150], inflammation [151] and thrombosis [11,35]. It is possible that the combination of phytochemicals as those occurring in the Mediterranean diet have amplified actions in comparison to sole compounds [152]. In fact, complex natural product mixtures synergistically target multiple networks involved in inflammatory and thrombosis [152]. The adoption of Mediterranean diet as a whole reduces PAF induced platelet aggregation in patients with 2 diabetes [153,154]. Moreover, it has been suggested to be a potentially protective diet against COVID-19 [10,155]. It is noted that the adoption of the Mediterranean Diet decreases length of stay and mortality in hospitalized patients >65 y of age [156,157], which is of interest in the era of COVID-19 and the challenges of health systems.

Several natural products which are intrinsic characteristics of the Mediterranean diet such as garlic, salvia and olive oil have been proposed as additional measures for the prevention and treatment of COVID-19 [158]. These and additional Mediterranean foods will be briefly presented and a special reference will be provided on their relation with PAF and its enzymes. It is noted that PAF has been inversely related to a healthy dietary pattern including legumes, vegetables, poultry and fish [34].

### 3.1. Olive Oil

Olive oil contains monounsaturated fatty acids and several microconstituents with antioxidant and anti-thrombotic action, such as polyphenols [159] and polar lipids [36]. Its anti-oxidant, anti-inflammatory and anti-thrombotic action render it a candidate food against COVID-19 [158]. It is noted that olive oil polar lipids act as PAF antagonists [36,160], and bioactive compounds have also been found in olive oil pomace and its byproducts [161]. From in vitro data it has been shown that olive oil polar lipids inhibit PAF-CPT which is a biosynthetic enzyme for PAF [142]. Moreover, lyso-PAF-AT has been negatively associated with a dietary pattern rich in olive oil and whole-wheat products as documented by our research team [34]. Lastly, the consumption of a yogurt enriched with PAF-inhibitors isolated from olive-oil by-products, led to attenuation of subclinical inflammation and platelet sensitivity to thrombotic stimuli in apparently healthy volunteers [162].

### 3.2. Fish

Fish have anti-inflammatory and anti-thrombotic properties, and they exert beneficial effects in the respiratory tract (see also omega-3 fatty acids) [159]. Indeed, fish has anti-aggregatory effects mediated by PAF inhibition [153,163,164] attributed to polar lipids, neutral lipids [165] and other lipids, such as gangliosides [166]. Moreover, from in vitro data it has been shown that fish polar lipids inhibit PAF-CPT [142]. Fish polar lipids retard atherosclerosis in rabbits by down-regulating PAF biosynthesis and up-regulating PAF catabolism [167]. Interestingly, the antibacterial properties of fish go hand in hand with their anti-PAF activity, suggesting that PAF antagonists and agonists in fish may also have antibacterial activity [168].

### 3.3. Honey

Detailed nutritional records of Cretan participants of the Seven Countries Study, highlight the presence of honey in their every-day diet [169] and Plato considered honey an essential component of a healthy diet [170]. Indeed, stingless bee honey has been found to inhibit TNF-α, IL-6 and interferon secretion from stimulated macrophages [171] and to reduce inflammation in animal models [172]. Honey has anti-bacterial properties due to its content of phenolic compounds, the production of hydrogen peroxide and other mechanisms such as osmosis [173]. Moreover, it has been suggested to have a role against COVID-19 epidemic [174,175], it has six compounds related to the receptor active site of COVID-19’s main protease according to a in silico approach [176] and is currently being tested in a clinical trial (clinical trial NCT04323345) [176]. It is noted that honey displays anti-thrombotic activity [177] and it especially acts as a PAF inhibitor [178]. In total, the anti-bacterial, the anti-thrombotic and anti-PAF effects of honey render it a potentially useful food against the COVID epidemic.

### 3.4. Milk and Yogurt

Dairy products constitute a principal source of vitamin D, which has been proposed to play a role in the fight against the COVID-19 epidemic [179,180,181]. It is noted that milk, yogurt and fermented milk products also contain PAF inhibitors [182,183,184], with goat yogurt presenting a more protective effect [185].

### 3.5. Plant Foods

Plant foods with antiviral properties have been recently reviewed as anti-COVID agents, as they prevent viral replication, enhance antibody production against influenza virus, and improve T-cell function [159]. A recent work reported the inhibition of COVID-19 with the use of molecular docking by plant terpenoids, such as Ginkgolide A [186], which is also one of the most potent PAF inhibitors [187]. Garlic and onion which are also used in many recipes of the Mediterranean diet [188] also contain PAF inhibitors [189,190]. Moreover, wild greens, which are rich in polyphenols have a postprandial anti-PAF effect [191]. Rice (Oryza sativa L.), traditionally used in several Mediterranean meals [188] may also be implicated in COVID-19, since rice bran policosanol extract has anti-aggregatory activity (although studies have researched only ADP-induced platelet aggregation and not PAF as an aggregatory agent) [192]. In addition, rice policosanol has been found to activate the nuclear factor erythroid 2-related factor 2 (Nrf-2) pathway[193], a molecular pathway playing a role in combating COVID-19 [194], which can also modulate PAF-acetylhydrolase transcription [195].

### 3.6. Wine and Its Products

Although wine is not recommended by national bodies as a means to fight coronavirus [8], the Mediterranean way of living and eating incorporates moderate wine consumption in its philosophy. A Mediterranean diet with moderate wine quantities could affect the pro-thrombotic status [196] and possibly the body’s response to a virus. As it has been documented by our group wine consumption reduces PAF-induced platelet aggregation [197] and specific wine varieties affect PAF biosynthetic enzymes [198] in the postprandial state. Moreover, several bioactive lipids have been isolated from wines that exhibit anti-PAF biological activity [199,200,201,202] and reduce the activity of its biosynthetic enzymes in monocytes [143]. Last but not least, bioactive compounds with anti-aggregatory have been also isolated from grape pomace extracts [203], which may render winery by-products useful for the production of functional foods.

## 4. Data from Clinical Trials

Since the results of clinical trials can be different from those obtained from in vitro studies a special reference is made to clinical trials regarding nutrient/foods and PAF metabolism (Table 1). As it can be seen, the majority of studies have focused on platelet aggregation [153,154,162,191,197,203,204,205,206,207] and/or PAF catabolic enzymes [108,206,208,209,210,211,212,213,214,215] and had promising results in both healthy subjects [108,154,162,197,198,206,207,216] and high-risk individuals [153,191,204,208,209,210,211,212,213,214,215,217,218,219,220,221,222]. Additionally, ongoing or recently finished clinical trials regarding CODIV-19 are displayed in Table 2. It is noted that only nutrients or foods which may modulate PAF and/or its enzymes are displayed. To our knowledge there is no ongoing trial with such nutrients and PAF measurement as an end point.

## 5. Hypothesis versus Epidemiological Data

The hypothesis of the protective effect of the Mediterranean Diet against COVID-19 should be regarded in parallel with epidemiological data. It can be argued that several Mediterranean countries, such as Italy and Spain had a high burden of the disease. It is difficult to make a safe assumption since the adherence of Southern European Mediterranean countries to the Mediterranean diet is generally considered rather moderate [228]. However, the adoption of the Mediterranean diet is lower in northern Italy than in the south of the country, which may in part explain the observed situation in Italy [229].

Moreover, dietary changes in the quarantine may account for some differences, since limited access to fresh foods may be observed, in favor of packaged foods, which have a longer shelf life. Indeed, in Italy, 37.3% of respondents changed their diet and physical activity levels but only 16.7% of them improved their habits [230]. Italian adolescents increased their intake of legumes, fruit, sweets, and fast food during quarantine and had no change in vegetables intake, while Spanish adolescents and the general population displayed more healthy changes in their diet [231,232].

In contrast, preliminary results from the COVIdiet in Greece, presented at the Webinar held by the Hellenic Dietetic Association have shown that participants improved their eating habits and reduced their consumption of fast food, especially those who were already more aware of the importance of a healthy diet. However, cooking increased, and the preparation and consumption of homemade sweets and pastries also increased increased [233].

## 6. Conclusions

In conclusion, there is no single food to prevent, heal, or treat coronavirus. Although the relationship between PAF and COVID-19 is not robust, a healthy diet containing PAF inhibitors may target both inflammation and thrombosis and prevent the deleterious effects of COVID-19. After completing our theoretical new approach on PAF and COVID-19, the next step is the experimental confirmation or not of the PAF–COVID-19 hypothesis.

## Figures and Tables

**Table 1:** Human clinical trials regarding the effects of foods/nutrients on PAF and its metabolism.

Nutrient/Food	Intervention	Volunteers	Age	Health Status	PAF Induced Platelet Aggregation	PAF Levels	PAF Biosynthetic Enzymes	PAF Catabolic Enzymes	Ref.
Vitamin D	15 weeks	*n* = 10 *n* = 9 (control)	56 ± 10 52 ± 13	Healthy				↓	[223]
Fish oils, omega-3									
Fish oil Olive oil	10 weeks	*n* = 15 (fish oil) *n* = 15 (olive oil)	61.9 ± 1.2	Peripheral vascular disease	↓ In the fish oil group ↑ in the olive oil group	no changes (measured in neutrophils)			[224]
Fish Fish oil (2 doses) Fish + fish oil placebo	12 weeks	*n* = 120 (for all groups)	30–60		↓ (not in the control group)				[204]
EPA + DHA omega-6	acute	*n* = 20		Psoriasis		↓ in n-3 Group ↑ in the n-6 group			[205]
omega-3 +atorvastatin placebo + atorvastatin	8 weeks	*n* =123 *n* = 122	56.1 ± 10.2	Hypertriglyceridemia				↓ (n-3 + atorvastatin vs. placebo + atorvastatin	[218]
EPA (2 doses) *	12 weeks	*n* = 702 (for all groups)	61 ± 10	Hypertriglyceridemia				↓	[225]
EPA *	12 weeks	*n* = 126 *n* = 120	60.2 ± 9.7 61.0 ± 9.9	Hypertriglyceridemia, high CRP				↓	[226]
EPA *	12 weeks	*n* = 19 (4 g) *n* = 30 (2 g) n = 36 (control)	68.2 ± 7.2 67.9 ± 8.3 68.0 ± 8.4	Hypertriglyceridemia, and chronic kidney disease				↓	[208]
EPA * (2 doses)	12 weeks	171 (2 g)_ 165 (4 g) 165(control)	Not reported	Hypertriglyceridemia, diabetes mellitus-2 and statin therapy				↓ (high dose)	[209]
EPA or DHA	6 weeks	*n* = 59 (for all groups, men)	61.2 ± 51.2	Hypertention and type 2 diabetes	no changes				[210]
omega-3	30 days	*n* = 54	30–80	angina				↓	[109]
EPA+ DHA 0/0.85/ 3.4 g/day	8 weeks	*n* = 25 (crossover)	44.3 ± 9.8	Hypertriglyceridemia				↓	[110]
EPA (2 g, 4 g) (control)		*n* = 77 (4 g) *n* = 76 (2 g) n = 76 (control)	52.9 ± 9.34	Hypertriglyceridemia				↓	[219]
omega-3 (2 g, 4 g) control	6 weeks	*n* = 209 (2 g) n= 207 (4 g) *n* = 211 (control	60.8 ± 9.6	Statin-treated patients with residual hypertriglyceridemia				↓	[220]
omega-3 esterified to glycerol or as ethyl esters	8 weeks	*n* = 120	62.4 ± 10.0	Hypertriglyceridemia				↓ With ethyl esters of n-3	[221]
omega-6 or omega -3 (parenteral nutrition)	10 days	*n* = 10 patients *n* = 8 healthy control	53.7 ± 13.8	Sepsis		↑in the n-3 group (baseline levels were suppressed)			[222]
omega-3 2 g, 3, 4 g	12 weeks	*n* = 100 (2 g) *n* = 101 (3 g) *n* - = 99 (4 g) n = 99 (control)	51.1 ± 9.8 51.2 ± 8.8 52.9 ± 10.9 50.8 ± 10.6	Hypertriglyceridemia				↓	[211]
EPA (2 g, 4 g) control	12 weeks	*n* = 215 (women)	~60 ± 10	Hypertriglyceridemia				↓	[212]
omega-3	3 months	*n* = 27 *n* = 35 (control)	62.3 ± 9.7 60.2 ± 10.8	Hypertension				no change	[213]
a-linolenic acid EPA+DHA	8 weeks	*n* = 20 ALA *n* = 20 EPA + DHA *n* - = 19 (control)	63.4 ± 8.2 62.1 ± 7.7 58.6 ± 6.3	Healthy				no change	
omega-3 (2 g, 6.6 g) control (olive oil)	12 weeks	*n* = 20 (2 g) *n* = 20 (6.6 g) *n* = 20 (control)	36.5 ± 11 37.0 ± 10 37.9 ± 10	Healthy				no change	[108]
olive oil (control) EPA 600 mg/day EPA 1800 mg/day, DHA 600 mg/day	6 weeks	*n* = 26 (control) *n* = 27 (600 mg EPA) *n* = 26 (1800 mg EPA) *n* = 28 (600 mg DHA)	52.2 ± 10.4 52.8 ± 11.6 52.2 ± 11.6 52.3 ± 12.6	Healthy				↓ high dose EPA	[217]
Mediterranean diet									
fast-food Mediterranean-type diet	4 weeks	*n* = 22 healthy *n* = 22 type 2 diabetes *n* = 22 control	56 ± 15	Healthy and with type 2 diabetes	↓ (not in the control group)				[154]
traditional Greek Mediterranean-type meals	28 days	*n* = 22 healthy *n* = 24 type 2 diabetes *n* = 22 type 2 diabetes -control	53 ± 12	Healthy and with type 2 diabetes	↓ (not in the control group)				[153]
Diet and exercise									
Diet and exercise	24 weeks	*n* = 22	44.0 ± 1.3	HIV				↓	[214]
substitution of whole grains and legumes for refined rice	12 weeks	*n* = 50 (whole grain) *n* = 49 (control)	56.3 ± 1.2 55.4 ± 1.5	Impaired fasting glucose, impaired glucose tolerance or newly diagnosed T2D				↓	[215]
Plants and plant extracts									
wild plant meals, namely, Reichardia picroides, Cynara cardunculus, Urospermum picroides and Chrysanthemum coronarium, and a control meal, which contained no wild plant	acute	*n* = 24	58.6 ± 11.3	Metabolic syndrome	↓ with the Urospermum picroides meal				[191]
plant extract supplement	8 weeks	*n* = 30 (supplement) *n* = 28 (control)	34.9 ± 5.8 (supplement) 32.9 ± 5.6 (control group)	Healthy	↓		no change	↑	[206]
ginkgolide mixture	acute	*n* = 6	25–35	Healthy	↓				[207]
Garlic extract	5 days	*n* = 14	20–55	Healthy	no change				[216]
Alcohol and wine									
Wine (Robola, Cabernet Sauvignon)	acute	*n* = 12	31.3 ± 4.3y	Healthy			↓lyso-PAf-AT ↓ PAF-CPT	no changes	[198]
Wine (Robola, Cabernet Sauvignon)	acute	*n* = 10	31.3 ± 4.3	Healthy	↓				[197]
Beer or alcohol-free	3 weeks	*n* = 11 lean *n* = 9 overweight	19 ± 2 21 ± 2	Healthy				no changes	[227]
Others									
Yogurt with bioactive ingredients from olive-oil by-products	8 weeks	*n* = 92	35–65	Healthy	↓				[162]

* Results from the same study (ANCHOR study). ↓: reduction; ↑: increase; EPA: Eicosapentaenoic acid; DHA: Docosahexaenoic acid; CRP: C-reactive protein; ALA: alpha-linolenic acid; AT:acetyltransferase, CPT: cholinephosphotransferase.

**Table 2:** Clinical trials regarding COVID-19 and foods or nutrients with anti-PAF actions.

Nutrient-Food	Quantity	Duration	Volunteers	Main Outcomes	Registration at www.clinicaltrials.gov
Vitamin C	10 g		400		NCT04584437
Vitamin C	10 g intravenously	72 hours	500	In-hospital mortality, length of stay, virus load	NCT04323514
Vitamin C and melatonin	1 g vitamin C 10 mg melatonin	14 days	150	Symptom severity	NCT04530539
Vitamin C and zinc	8 g vitamin C or 50 mg zinc or 8 g vitamin C + 50 mg zinc	28 days	520	Symptom duration	NCT04342728
Vitamin C, vitamin D, zinc	Not reported	12 weeks	600	Rate of recover, symptoms,	NCT04334512
Vitamin C, vitamin D, zinc, B12	Vitamin C 28 g intravenously zinc Citrate 30 mg Vitamin D3 5000 IU daily Vitamin B12 500 ug	7–14 days	200	Symptoms, length of stay	NCT04395768
Vitamin C, vitamin D, zinc	Not reported	14 weeks	600 medical workers	Prevention of COVID-19 symptoms	NCT04335084
Vitamin D	9600 IU/day on days 1 and 2, and 3200 IU/day on days 3 through 28	28 days	2700 participants with newly diagnosed COVID-19	Hospitalization or death in index cases, self-reported disease severity in index cases time to hospitalization or death in index cases, ICU admission/ventilation support in index cases, SARS-CoV-2 infection in close household contacts, self-reported disease severity in close household contacts	NCT04536298
Vitamin D	50,000 IU/week	8 weeks	100	Cytokine levels	NCT04476745
Vitamin D	200,000 IU on admission		240	Length of hospitalization, Number of cases admitted to Intensive Care Unit, Length of use of mechanic ventilator inflammatory markers, vitamin D,	NCT04449718
Vitamin D	10,000 IU bolus dose followed by 10,000 IU once a week	16 weeks	2414 health care workers	Distribution of disease severity, disease severity	NCT04483635
Vitamin D	800 IU 3200 IU	6 m	6200 individuals with 25-hydroxyvitamin D level <75 nmol/L	Acute respiratory infection, COVID-19 diagnosis	NCT04579640
Vitamin D	10,000 IU/day (age 18–69 years) or 15,000 IU/day (age 70+) 2 w: if vitamin D <30 ng/mL, continue the dosage for 3 more weeks. If vitamin D: 30–49 ng/mL, continue at a dosage of 5000 IU/day. If vitamin D >50 ng/mL, stop supplementation.	6 weeks	41	Vitamin D, severity of COVID-19 symptoms	NCT04407286
Vitamin D	6000 IU 6000 IU + 20,000 IU vitamin D3 daily for 3 days	12 m	140	Vitamin D, Change in SARS-CoV-2 antibody titers, inflammatory markers	NCT04482673
Vitamin D	5000 IU)	9 m	2099 hospital workers	Respiratory tract infections	NCT04596657
vitamin D and zinc	2000 IU 30 mg	2 m	3140	Survival rate	NCT04351490
vitamin D and zinc	180,000 international units (IU) 40 mg of zinc	8 weeks	700	Time to recover, all-cause mortality, symptoms, levels of vitamins	NCT04641195
Omega-3	300 mg of omega3-FA	8 weeks	100	Serum ACE levels, serum ACE2 levels, lipid profile	NCT04658433
Fish oil	wild salmon and fish oil complex 1 g, 300 mg omega-3	8 weeks	100	Cytokine levels, lipid profile, glucose levels	NCT04483271
Fish oil	Cod liver oil: 5 mL (Contains: 10 ug of vitamin D, 1.2 g of long-chained n-3 polyunsaturated fatty acids (DHA 0.6 g and EPA 0.4 g), 250 ug vitamin A and 10 mg vitamin E).	6 m	80,000	Number of participants diagnosed with serious Covid-19, self-reported airway infection, hospitalization, infections	NCT04609423
Zinc, Quercetin, Bromelain and Vitamin C	zinc 50 mg vitamin C 1000 mg	5–10 days	60	Time to hospital discharge serum zinc Time of negativization of COVID-PCR	NCT04468139
Zinc, vitamin C	Zinc 220 mg vitamin C 1 g	10 days	50	Symptoms reduction time frame, severity of symptoms	NCT04558424
Zinc	high dose Zinc supplementation in combination with copper, vitamin C/E and beta-carotene vs. low dose zinc and multivitamin supplement	3 m	4500	Hospitalization, Illness without hospitalization, mortality	NCT04551339
Anti-inflammatory/antioxidant supplement	vitamin A (as β-carotene) 500 ug, Vitamin C 250 mg, vitamin E 90 mg, Selenium 15 ug, Zinc 7.5 mg.	14 days	40	Nutritional risk, inflammatory indices, ferritin, anthropometry etc.	NCT04323228
Quercetin	500 mg	30 days	200	Survival time, Length of stay in hospital, days of mechanical ventilation, blood exams etc.	NCT04578158
Licorice	250 mg standardized extract (25% Glycyrrhizin - 62.5 mg)	10 days	70	Number of people recovering from COVID-19, mechanical support, hospital stay	NCT04487964
Plant polyphenol	Plant polyphenol +Vitamin D3 100,000 IU on day 1	15 days	200	Hospitalization rates for COVID-19	NCT04400890
Herbal extract (Cretan IAMA)	1 mL/day Thymbra 59 capitata (L.) Cav., Origanum dictamnus L., Salvia fruticosa Mill. in extra virgin olive oil	2 weeks	20	Symptom resolution	NCT04705753
Honey	1 gm/kg/day	14 days	1000	Rate of recovery, resolution of lung inflammation	NCT04323345

ACE: Angiotensin converting enzyme.

## Data Availability

Not applicable.

## Abbreviations

PAF	platelet-activating factor
PAF-CPT	dithiothreitol-insensitive cholinephosphotransferase
CDP-choline	cytidine diphosphate-choline
Lp-PLA_2_	lipoprotein associated phospholipase A_2_
ACE2	angiotensin converting enzyme 2
TLR	Toll-like receptor
EPA	Eicosapentaenoic acid
DHA	Docosahexaenoic acid
CRP	C-reactive protein
ALA	alpha-linolenic acid
AT	acetyltransferase

## References

[1] Guan W.J., Ni Z.Y., Hu Y., Liang W.H., Ou C.Q., He J.X., Liu L., Shan H., Lei C.L., Hui D.S.C. (2020). Clinical Characteristics of coronavirus disease 2019 in China. N. Engl. J. Med..

[2] Demopoulos C., Antonopoulou S., Theocharides T. (2002). CoVid-19, microthromboses, inflammation and platelet activating factor (PAF). Biofactors.

[3] Zhao X., Li Y., Ge Y., Shi Y., Lv P., Zhang J., Fu G., Zhou Y., Jiang K., Lin N. (2020). Evaluation of nutrition risk and its association with mortality risk in severely and critically Ill COVID-19 Patients. J. Parenter. Enter. Nutr..

[4] British Dietetic Association (2020). COVID-19/Coronavirus—Advice for the General Public. https://www.bda.uk.com/resource/covid-19-corona-virus-advice-for-the-general-public.html.

[5] Caccialanza R., Laviano A., Lobascio F., Montagna E., Bruno R., Ludovisi S., Corsico A.G., Di Sabatino A., Belliato M., Calvi M. (2020). Early nutritional supplementation in non-critically ill patients hospitalized for the 2019 novel coronavirus disease (COVID-19): Rationale and feasibility of a shared pragmatic protocol. Nutrition.

[6] Cintoni M., Rinninella E., Annetta M.G., Mele M.C. (2020). Nutritional management in hospital setting during SARS-CoV-2 pandemic: A real-life experience. Eur. J. Clin. Nutr..

[7] Carr A.C. (2020). A new clinical trial to test high-dose vitamin C in patients with COVID-19. Crit. Care.

[8] Food and Agriculture Organization of the United Nations (2020). Maintaining a Healthy Diet during the COVID-19 Pandemic. http://www.fao.org/3/ca8380en/CA8380EN.pdf.

[9] Kelesidis T., Papakonstantinou V., Detopoulou P., Fragopoulou E., Chini M., Lazanas M.C., Antonopoulou S. (2015). The role of platelet-activating factor in chronic inflammation, immune activation, and comorbidities associated with hiv infection. Aids Rev..

[10] Zabetakis I., Lordan R., Norton C., Tsoupras A. (2020). COVID-19: The Inflammation link and the role of nutrition in potential mitigation. Nutrients.

[11] Nomikos T., Fragopoulou E., Antonopoulou S., Panagiotakos D.B. (2018). Mediterranean diet and platelet-activating factor; a systematic review. Clin. Biochem..

[12] Tsoupras A., Lordan R., Zabetakis I. (2020). Thrombosis and COVID-19: The Potential role of nutrition. Front. Nutr..

[13] Theoharides T.C., Antonopoulou S., Demopoulos C.A. (2020). Coronavirus 2019, Microthromboses, and platelet activating factor. Clin. Ther..

[14] Demopoulos C.A., Pinckard R.N., Hanahan D.J. (1979). Platelet-activating factor. Evidence for 1-O-alkyl-2-acetyl-sn-glyceryl-3-phosphorylcholine as the active component (a new class of lipid chemical mediators). J. Biol. Chem..

[15] Ashraf M.A., Nookala V. (2020). Biochemistry, Platelet activating factor. StatPearls.

[16] Lordan R., Tsoupras A., Zabetakis I., Demopoulos C.A. (2019). Forty years since the structural elucidation of platelet-activating factor (PAF): Historical, Current, and future research perspectives. Molecules.

[17] Detopoulou P., Nomikos T., Fragopoulou E., Chrysohoou C., Antonopoulou S. (2013). Platelet activating factor in heart failure: Potential role in disease progression and novel target for therapy. Curr. Heart Fail. Rep..

[18] Ivanov A.I., Patel S., Kulchitsky V.A., Romanovsky A.A. (2003). Platelet-Activating factor: A Previously unrecognized mediator of fever. J. Physiol..

[19] Kawaguchi H., Sawa H., Yasuda H. (1990). Mechanism of increased angiotensin-converting enzyme activity stimulated by platelet-activating factor. Biochim. Biophys. Acta Bioenerg..

[20] Datta P.K., Liu F., Fischer T., Rappaport J., Qin X. (2020). SARS-CoV-2 pandemic and research gaps: Understanding SARS-CoV-2 interaction with the ACE2 receptor and implications for therapy. Theranostics.

[21] Yan B., Chu H., Yang D., Sze K.-H., Lai P.-M., Yuan S., Shuai H., Wang Y., Kao R.Y.T., Chan J.F.-W. (2019). Characterization of the Lipidomic profile of human coronavirus-infected cells: Implications for Lipid metabolism remodeling upon coronavirus replication. Viruses.

[22] Marathe G. (2001). Oxidized LDL Contains inflammatory PAF-Like phospholipids. Trends Cardiovasc. Med..

[23] Liapikos T.A., Antonopoulou S., Karabina S.-A.P., Tsoukatos D.C.A., Demopoulos C.A., Tselepis A.D. (1994). Platelet-activating factor formation during oxidative modification of low-density lipoprotein when PAF-acetylhydrolase has been inactivated. Biochim. Biophys. Acta Lipids Lipid Metab..

[24] Imai Y., Kuba K., Neely G.G., Yaghubian-Malhami R., Perkmann T., van Loo G., Ermolaeva M., Veldhuizen R., Leung Y.H.C., Wang H. (2008). Identification of Oxidative stress and toll-like receptor 4 signaling as a key pathway of acute lung injury. Cell.

[25] Yi L., Zhang J., Zhong J., Zheng Y. (2020). Elevated Levels of platelet activating factor and its acetylhydrolase indicate high risk of kawasaki disease. J. Interferon Cytokine Res..

[26] Muehlmann L.A., Michelotto P.V., Nunes E.A., Grando F.C.C., da Silva F.T., Nishiyama A. (2012). PAF increases phagocytic capacity and superoxide anion production in equine alveolar macrophages and blood neutrophils. Res. Vet. Sci..

[27] Karagiorga G., Nakos G., Galiatsou E., Lekka M.E. (2005). Biochemical parameters of bronchoalveolar lavage fluid in fat embolism. Intensive Care Med..

[28] Caplan M.S., Hsueh W., Sun X., Gidding S.S., Hageman J.R. (1990). Circulating plasma platelet activating factor in persistent pulmonary hypertension of the newborn. Am. Rev. Respir. Dis..

[29] Trimoreau F., François B., Desachy A., Besse A., Vignon P., Denizot Y. (2000). Platelet-activating factor acetylhydrolase and haemophagocytosis in the sepsis syndrome. Mediat. Inflamm..

[30] Falk S., Göggel R., Heydasch U., Brasch F., Muller K.-M., Wendel A., Uhlig S. (1999). Quinolines Attenuate paf-induced pulmonary pressor responses and edema formation. Am. J. Respir. Crit. Care Med..

[31] Muñoz-Cano R.M., Casas-Saucedo R., Santiago A.V., Bobolea I., Ribó P., Mullol J. (2019). Platelet-Activating factor (PAF) in Allergic rhinitis: Clinical and therapeutic implications. J. Clin. Med..

[32] Tsantila N., Tsoupras A.B., Fragopoulou E., Antonopoulou S., Iatrou C., Demopoulos C.A. (2010). In Vitro and in vivo effects of statins on platelet-activating factor and its metabolism. Angiology.

[33] Tsoupras A.B., Chini M., Tsogas N., Fragopoulou E., Nomikos T., Lioni A., Mangafas N., Demopoulos C.A., Antonopoulou S., Lazanas M.C. (2008). Anti-platelet-activating factor effects of highly active antiretroviral therapy (HAART): A New insight in the drug therapy of HIV infection?. AIDS Res. Hum. Retrovir..

[34] Detopoulou P., Fragopoulou E., Nomikos T., Yannakoulia M., Stamatakis G., Panagiotakos D.B., Antonopoulou S. (2014). The relation of diet with PAF and its metabolic enzymes in healthy volunteers. Eur. J. Nutr..

[35] Detopoulou P., Demopoulos C.A., Karantonis H.C., Antonopoulou S. (2015). Mediterranean diet and its protective mechanisms against cardiovascular disease: An insight into Platelet Activating Factor (PAF) and diet interplay. Ann. Nutr. Disord. Ther..

[36] Tsantila N., Karantonis H.C., Perrea D.N., Theocharis S.E., Iliopoulos D.G., Antonopoulou S., Demopoulos C.A. (2007). Antithrombotic and Antiatherosclerotic Properties of olive oil and olive pomace polar extracts in rabbits. Mediat. Inflamm..

[37] Fernández-Quintela A., Milton-Laskíbar I., Trepiana J., Gómez-Zorita S., Kajarabille N., Léniz A., González M., Portillo M.P. (2020). Key Aspects in nutritional management of COVID-19 Patients. J. Clin. Med..

[38] Gasmi A., Tippairote T., Mujawdiya P.K., Peana M., Menzel A., Dadar M., Benahmed A.G., Bjørklund G. (2020). Micronutrients as immunomodulatory tools for COVID-19 management. Clin. Immunol..

[39] Chew B.P., Park J.S. (2004). Carotenoid action on the immune response. J. Nutr..

[40] Sezavar H., Saboor-Yaraghi A.-A., Salehi E., Mottaghi A. (2014). Whether vitamin A supplementation is effective in T-bet and IFN-ɣ gene expression reduction?. Immunol. Investig..

[41] Timoneda J., Rodríguez-Fernández L., Zaragozá R., Marín M., Cabezuelo M., Torres L., Viña J., Barber T. (2018). Vitamin A deficiency and the lung. Nutrients.

[42] Mutoh H., Fukuda T., Kitamaoto T., Masushige S., Sasaki H., Shimizu T., Kato S. (1996). Tissue-specific response of the human platelet-activating factor receptor gene to retinoic acid and thyroid hormone by alternative promoter usage. Proc. Natl. Acad. Sci. USA.

[43] Bazan N.G., Fletcher B.S., Herschman H.R., Mukherjee P.K. (1994). Platelet-activating factor and retinoic acid synergistically activate the inducible prostaglandin synthase gene. Proc. Natl. Acad. Sci. USA.

[44] Claar D., Hartert T.V., Peebles R.S. (2014). The role of prostaglandins in allergic lung inflammation and asthma. Expert Rev. Respir. Med..

[45] Tsimikas S., Willeit J., Knoflach M., Mayr M., Egger G., Notdurfter M., Witztum J.L., Wiedermann C.J., Xu Q., Kiechl S. (2008). Lipoprotein-associated phospholipase A2 activity, ferritin levels, metabolic syndrome, and 10-year cardiovascular and non-cardiovascular mortality: Results from the Bruneck study. Eur. Heart J..

[46] Ito S., Camussi G., Tetta C., Milgrom F., Andres G. (1984). Hyperacute renal allograft rejection in the rabbit. The role of platelet-activating factor and of cationic proteins derived from polymorphonuclear leukocytes and from platelets. Lab. Investig..

[47] Carr A.C., Maggini S. (2017). Vitamin C and Immune function. Nutrients.

[48] Shakoor H., Feehan J., Al Dhaheri A.S., Ali H.I., Platat C., Ismail L.C., Apostolopoulos V., Stojanovska L. (2021). Immune-boosting role of vitamins D, C, E, zinc, selenium and omega-3 fatty acids: Could they help against COVID-19?. Maturitas.

[49] Hemilä H., Louhiala P. (2013). Vitamin C for preventing and treating pneumonia. Cochrane Database Syst. Rev..

[50] Australian Governement, Department of Health (2020). No Evidence to Support Intravenous High-Dose Vitamin C in the Management of COVID-19.

[51] Tousoulis D., Antoniades C., Tountas C., Bosinakou E., Kotsopoulou M., Toutouzas P., Stefanadis C. (2003). Vitamin C affects thrombosis/ fibrinolysis system and reactive hyperemia in patients with type 2 diabetes and coronary artery disease. Diabetes Care.

[52] Spittle C.R. (1973). Vitamin C and deep vein thrombosis. Lancet.

[53] Lloberas N., Torras J., Herrero-Fresneda I., Cruzado J.M., Riera M., Hurtado I., Grinyó J.M. (2002). Postischemic renal oxidative stress induces an inflammatory response through PAF and oxidized phospholipids: Prevention by antioxidant treatment. FASEB J..

[54] Lewis M.S., Whatley R.E., Cain P., McIntyre T.M., Prescott S.M., Zimmerman G.A. (1988). Hydrogen peroxide stimulates the synthesis of platelet-activating factor by endothelium and induces endothelial cell-dependent neutrophil adhesion. J. Clin. Investig..

[55] Verouti S.N., Fragopoulou E., Karantonis H.C., Dimitriou A.A., Tselepis A.D., Antonopoulou S., Nomikos T., Demopoulos C.A. (2011). PAF effects on MCP-1 and IL-6 secretion in U-937 monocytes in comparison with OxLDL and IL-1β effects. Atherosclerosis.

[56] Hewitt J., Carter B., Vilches-Moraga A., Quinn T.J., Braude P., Verduri A., Pearce L., Stechman M., Short R., Price A. (2020). The effect of frailty on survival in patients with COVID-19 (COPE): A multicentre, european, observational cohort study. Lancet Public Health.

[57] Soysal P., Isik A.T., Carvalho A.F., Fernandes B.S., Solmi M., Schofield P., Veronese N., Stubbs B. (2017). Oxidative stress and frailty: A systematic review and synthesis of the best evidence. Maturitas.

[58] Bourbour F., Dahka S.M., Gholamalizadeh M., Akbari M.E., Shadnoush M., Haghighi M., Taghvaye-Masoumi H., Ashoori N., Doaei S. (2020). Nutrients in prevention, treatment, and management of viral infections; special focus on Coronavirus. Arch. Physiol. Biochem..

[59] Martineau A.R., Jolliffe D.A., Hooper R.L., Greenberg L., Aloia J.F., Bergman P., Dubnov-Raz G., Esposito S., Ganmaa D., Ginde A.A. (2017). Vitamin D supplementation to prevent acute respiratory tract infections: Systematic review and meta-analysis of individual participant data. BMJ.

[60] Hu Y.-C., Wang W.-W., Jiang W.-Y., Li C.-Q., Guo J.-C., Xun Y. (2019). Low vitamin D levels are associated with high viral loads in patients with chronic hepatitis B: A systematic review and meta-analysis. BMC Gastroenterol..

[61] Chun R.F., Liu N.Q., Lee T., Schall J.I., Denburg M.R., Rutstein R.M., Adams J.S., Zemel B.S., Stallings V.A., Hewison M. (2015). Vitamin D supplementation and antibacterial immune responses in adolescents and young adults with HIV/AIDS. J. Steroid Biochem. Mol. Biol..

[62] Ilie P.C., Stefanescu S., Smith L. (2020). The role of vitamin D in the prevention of coronavirus disease 2019 infection and mortality. Aging Clin. Exp. Res..

[63] Hastie C.E., Mackay D.F., Ho F.K., Celis-Morales C.A., Katikireddi S.V., Niedzwiedz C.L., Jani B.D., Welsh P., Mair F.S., Gray S.R. (2020). Vitamin D concentrations and COVID-19 infection in UK Biobank. Diabetes Metab. Syndr. Clin. Res. Rev..

[64] D’Avolio A., Avataneo V., Manca A., Cusato J., De Nicolò A., Lucchini R., Keller F., Cantù M. (2020). 25-Hydroxyvitamin D Concentrations are lower in patients with positive PCR for SARS-CoV-2. Nutrients.

[65] Im J.H., Je Y.S., Baek J., Chung M.-H., Kwon H.Y., Lee J.-S. (2020). Nutritional status of patients with COVID-19. Int. J. Infect. Dis..

[66] Narahara H., Miyakawa I., Johnston J.M. (1995). The inhibitory effect of 1,25-dihydroxyvitamin D3 on the secretion of platelet-activating factor acetylhydrolase by human decidual macrophages. J. Clin. Endocrinol. Metab..

[67] Verouti S.N., Tsoupras A.B., Alevizopoulou F., Demopoulos C.A., Iatrou C. (2013). Paricalcitol effects on activities and metabolism of platelet activating factor and on inflammatory cytokines in hemodialysis patients. Int. J. Artif. Organs.

[68] Mohammad S., Mishra A., Ashraf M.Z. (2019). Emerging role of vitamin d and its associated molecules in pathways related to pathogenesis of thrombosis. Biomolecules.

[69] Coquette A., Vray B., Vanderpas J. (1986). Role of vitamin E in the protection of the resident macrophage membrane against oxidative damage. Arch. Int. Physiol. Biochim..

[70] Mileva M., Bakalova R., Tancheva L., Galabov A., Ribarov S. (2002). Effect of vitamin E supplementation on lipid peroxidation in blood and lung of influenza virus infected mice. Comp. Immunol. Microbiol. Infect. Dis..

[71] Hayek M.G., Taylor S.F., Bender B.S., Han S.N., Meydani M., Smith D.E., Eghtesada S., Meydani S.N. (1997). Vitamin E Supplementation decreases lung virus titers in mice infected with influenza. J. Infect. Dis..

[72] Galabov A.S., Mileva M., Simeonova L., Gegova G. (2015). Combination activity of neuraminidase inhibitor oseltamivir and α-tocopherol in influenza virus A (H3N2) infection in mice. Antivir. Chem. Chemother..

[73] Saboori S., Shab-Bidar S., Speakman J.R., Rad E.Y., Djafarian K. (2015). Effect of vitamin E supplementation on serum C-reactive protein level: A meta-analysis of randomized controlled trials. Eur. J. Clin. Nutr..

[74] Wang J.-Z., Zhang R.-Y., Bai J. (2020). An anti-oxidative therapy for ameliorating cardiac injuries of critically ill COVID-19-infected patients. Int. J. Cardiol..

[75] Fukuzawa K., Kurotori Y., Tokumura A., Tsukatani H. (1989). Vitamin E. deficiency increases the synthesis of platelet-activating factor (PAF) in rat polymorphonuclear leucocytes. Lipids.

[76] Akada S., Iioka H., Moriyama I., Hisanaga H., Morimoto K., Ichijo M. (1991). The role of vitamin E during pregnancy—anti-platelet aggregation activity of alpha-tocopherol. Nihon Sanka Fujinka Gakkai Zasshi.

[77] Violi F., Pratico D., Ghiselli A., Alessandri C., Iuliano L., Cordova C., Balsano F. (1990). Inhibition of cyclooxygenase-independent platelet aggregation by low vitamin E concentration. Atherosclerosis.

[78] Antonopoulou S., Demopoulos C. (1997). On the mediterranean diet. INFORM.

[79] Kakishita E., Suehiro A., Oura Y., Nagai K. (1990). Inhibitory effect of vitamin E (α-tocopherol) on spontaneous platelet aggregation in whole blood. Thromb. Res..

[80] Balestrieri M.L., De Prisco R., Nicolaus B., Pari P., Moriello V., Strazzullo G., Iorio E.L., Servillo L., Balestrieri C. (2004). Lycopene in association with α-tocopherol or tomato lipophilic extracts enhances acyl-platelet-activating factor biosynthesis in endothelial cells during oxidative stress. Free. Radic. Biol. Med..

[81] Larkin E.K., Gao Y.-T., Gebretsadik T., Hartman T.J., Wu P., Wen W., Yang G., Bai C., Jin M., Roberts L.J. (2015). New risk factors for adult-onset incident asthma. A nested case–control study of host antioxidant defense. Am. J. Respir. Crit. Care Med..

[82] Rainwater D.L., Mahaney M.C., VandeBerg J.L., Wang X.L. (2007). Vitamin E dietary supplementation significantly affects multiple risk factors for cardiovascular disease in baboons. Am. J. Clin. Nutr..

[83] Silbert P.L., Leong L.L.L., Sturm M.J., Strophair J., Taylor R.R. (1990). Short term vitamin e supplementation has no effect on platelet function, plasma phospholipase a2and lyso-paf in male volunteers. Clin. Exp. Pharmacol. Physiol..

[84] Kieliszek M., Lipinski B. (2020). Selenium supplementation in the prevention of coronavirus infections (COVID-19). Med. Hypotheses.

[85] Avery J.C., Hoffmann P.R. (2018). Selenium, Selenoproteins, and Immunity. Nutrients.

[86] Huang Z., Rose A.H., Hoffmann P.R. (2012). The role of selenium in inflammation and immunity: From Molecular mechanisms to therapeutic opportunities. Antioxid. Redox Signal..

[87] Yu L., Sun L., Nan Y., Zhu L.Y. (2010). Protection from H1N1 Influenza virus infections in mice by supplementation with selenium: A Comparison with selenium-deficient mice. Biol. Trace Elem. Res..

[88] Norton R.L., Hoffmann P.R. (2012). Selenium and asthma. Mol. Asp. Med..

[89] Zhang J., Taylor E.W., Bennett K., Saad R., Rayman M.P. (2020). Association between regional selenium status and reported outcome of COVID-19 cases in China. Am. J. Clin. Nutr..

[90] Cao Y.-Z., Cohen Z.S., Weaver J.A., Sordillo L.M. (2001). Selenium modulates 1-O-Alkyl-2-Acetyl-sn-Glycero-3-Phosphocholine (PAF) Biosynthesis in bovine aortic endothelial cells. Antioxid. Redox Signal..

[91] Hampel G., Watanabe K., Weksler B.B., Jaffe E.A. (1989). Selenium deficiency inhibits prostacyclin release and enhances production of platelet activating factor by human endothelial cells. Biochim. Biophys. Acta Lipids Lipid Metab..

[92] Kaur H.D., Bansal M.P. (2009). Studies on HDL associated enzymes under experimental hypercholesterolemia: Possible modulation on selenium supplementation. Lipids Health Dis..

[93] Ricetti M.M., Guidi G.C., Tecchio C., Bellisola G., Rigo A., Perona G. (1999). Effects of sodium selenite on in vitro interactions between platelets and endothelial cells. Int. J. Clin. Lab. Res..

[94] Weill P., Plissonneau C., Legrand P., Rioux V., Thibault R. (2020). May omega-3 fatty acid dietary supplementation help reduce severe complications in Covid-19 patients?. Biochimie.

[95] Sharma S., Chhibber S., Mohan H., Sharma S. (2013). Dietary supplementation with omega-3 polyunsaturated fatty acids ameliorates acute pneumonia induced by *Klebsiella pneumoniaein* BALB/c mice. Can. J. Microbiol..

[96] Hinojosa C.A., Gonzalez-Juarbe N., Rahman M., Fernandes G., Orihuela C.J., I Restrepo M.I. (2020). Omega-3 fatty acids in contrast to omega-6 protect against pneumococcal pneumonia. Microb. Pathog..

[97] Schwerbrock N.M.J., Karlsson E.A., Shi Q., Sheridan P.A., Beck M.A. (2009). Fish oil-fed mice have impaired resistance to influenza infection. J. Nutr..

[98] Byleveld P.M., Pang G.T., Clancy R.L., Roberts D.C.K. (1999). Fish Oil feeding delays influenza virus clearance and impairs production of interferon-γ and virus-specific Immunoglobulin A in the Lungs of mice. J. Nutr..

[99] DeFilippis A.P., Rai S.N., Cambon A., Miles R., Jaffe A.S., Moser A.B., Jones R.O., Bolli R., Schulman S.P. (2014). Fatty acids and TxA2 generation, in the absence of platelet-COX-1 activity. Nutr. Metab. Cardiovasc. Dis..

[100] Oh-Hashi K., Takahashi T., Watanabe S., Kobayashi T., Okuyama H. (1997). Possible mechanisms for the differential effects of high linoleate safflower oil and high α-linolenate perilla oil diets on platelet-activating factor production by rat polymorphonuclear leukocytes. J. Lipid Mediat. Cell Signal..

[101] Mayer K., Merfels M., Muhly-Reinholz M., Gokorsch S., Rosseau S., Lohmeyer J., Schwarzer N., Krüll M., Suttorp N., Grimminger F. (2002). ω-3 Fatty acids suppress monocyte adhesion to human endothelial cells: Role of endothelial PAF generation. Am. J. Physiol. Circ. Physiol..

[102] Shikano M., Masuzawa Y., Yazawa K. (1993). Effect of docosahexaenoic acid on the generation of platelet-activating factor by eosinophilic leukemia cells, Eol-1. J. Immunol..

[103] Weber C., Aepfelbacher M., Lux I., Zimmer B., Weber P.C. (1991). Docosahexaenoic acid inhibits PAF and LTD4 stimulated [Ca2+]i-increase in differentiated monocytic U937 cells. Biochim. Biophys. Acta Bioenerg..

[104] Sirivongrangson P., Kulvichit W., Payungporn S., Pisitkun T., Chindamporn A., Peerapornratana S., Pisitkun P., Chitcharoen S., Sawaswong V., Worasilchai N. (2020). Endotoxemia and circulating bacteriome in severe COVID-19 patients. Intensive Care Med. Exp..

[105] Oh-Hashi K., Takahashi T., Tanabe A., Watanabe S., Okuyama H. (1999). Dietary α-linolenate suppresses endotoxin-induced platelet-activating factor production in rat kidney. Lipids.

[106] Akisu M., Huseyinov A., Baka M., Yalaz M., Kultursay N. (2002). The effect of dietary supplementation with n-3 polyunsaturated fatty acids on the generation of platelet-activating factor and leukotriene B4 in hypoxic–ischemic brain in young mice. Prostaglandins Leukot. Essent. Fat. Acids.

[107] Schmidt E.B., Koenig W., Khuseyinova N., Christensen J.H. (2008). Lipoprotein-associated phospholipase A2 concentrations in plasma are associated with the extent of coronary artery disease and correlate to adipose tissue levels of marine n-3 fatty acids. Atherosclerosis.

[108] Pedersen M.W., Koenig W., Christensen J.H., Schmidt E.B. (2009). The effect of marine n-3 fatty acids in different doses on plasma concentrations of Lp-PLA2 in healthy adults. Eur. J. Nutr..

[109] Gajos G., Zalewski J., Mostowik M., Konduracka E., Nessler J., Undas A. (2014). Polyunsaturated omega-3 fatty acids reduce lipoprotein-associated phospholipase A2 in patients with stable angina. Nutr. Metab. Cardiovasc. Dis..

[110] Skulas-Ray A.C., Alaupovic P., Kris-Etherton P.M., West S.G. (2015). Dose-response effects of marine omega-3 fatty acids on apolipoproteins, apolipoprotein-defined lipoprotein subclasses, and Lp-PLA2 in individuals with moderate hypertriglyceridemia. J. Clin. Lipidol..

[111] Fragopoulou E., Detopoulou P., Alepoudea E., Nomikos T., Kalogeropoulos N., Antonopoulou S. (2021). Associations between red blood cells fatty acids, desaturases and metabolism of Platelet Activating Factor in healthy volunteers. Prostaglandins Leukotrienes Essential Fatty Acids.

[112] Te Velthuis A.J.W.T., van den Worm S.H.E., Sims A.C., Baric R.S., Snijder E.J., van Hemert M.J. (2010). Zn2+ Inhibits coronavirus and arterivirus RNA Polymerase activity in vitro and zinc ionophores block the replication of these viruses in cell culture. PLoS Pathog..

[113] Gombart A.F., Pierre A., Maggini S. (2020). A review of micronutrients and the immune system–working in harmony to reduce the risk of infection. Nutrients.

[114] Walker C.F., Black R.E. (2004). Zinc and the risk for infectious disease. Annu. Rev. Nutr..

[115] Kiabi F.H., Alipour A., Darvishi-Khezri H., Aliasgharian A., Zeydi A.E. (2017). Zinc supplementation in adult mechanically ventilated trauma patients is associated with decreased occurrence of ventilator-associated pneumonia: A secondary analysis of a prospective, observational study. Indian J. Crit. Care Med..

[116] Xue J., Moyer A., Peng B., Wu J., Hannafon B.N., Ding W.-Q. (2014). Chloroquine is a zinc ionophore. PLoS ONE.

[117] Johnson M.A., Fischer J.G., Kays S.E. (1992). Is copper an antioxidant nutrient?. Crit. Rev. Food Sci. Nutr..

[118] Bonham M., O’Connor J.M., Hannigan B.M., Strain J.J. (2002). The immune system as a physiological indicator of marginal copper status?. Br. J. Nutr..

[119] Besold A.N., Culbertson E.M., Culotta V.C. (2016). The Yin and Yang of copper during infection. JBIC J. Biol. Inorg. Chem..

[120] De Silva A., Atukorala S., Weerasinghe I., Ahluwalia N. (2003). Iron supplementation improves iron status and reduces morbidity in children with or without upper respiratory tract infections: A randomized controlled study in Colombo, Sri Lanka. Am. J. Clin. Nutr..

[121] Neves J., Haider T., Gassmann M., Muckenthaler M.U. (2019). Iron homeostasis in the lungs—a balance between health and disease. Pharmaceuticals.

[122] Liu W., Zhang S., Nekhai S., Liu S. (2020). Depriving Iron supply to the virus represents a promising adjuvant therapeutic against viral survival. Curr. Clin. Microbiol. Rep..

[123] Mammadova-Bach E., Braun A. (2019). Zinc homeostasis in platelet-related diseases. Int. J. Mol. Sci..

[124] Tsoupras A.B., Roulia M., Ferentinos E., Stamatopoulos I., Demopoulos C.A., Kyritsis P. (2010). Structurally diverse metal coordination compounds, bearing imidodiphosphinate and diphosphinoamine ligands, as potential inhibitors of the platelet activating factor. Bioinorg. Chem. Appl..

[125] Papakonstantinou V.D., Lagopati N., Tsilibary E.C., Demopoulos C.A., Philippopoulos A.I. (2017). A Review on platelet activating factor inhibitors: Could a new class of potent metal-based anti-inflammatory drugs induce anticancer properties?. Bioinorg. Chem. Appl..

[126] Wykle R.L., Malone B., Snyder F. (1980). Enzymatic synthesis of 1-alkyl-2-acetyl-sn-glycero-3-phosphocholine, a hypotensive and platelet-aggregating lipid. J. Biol. Chem..

[127] Ambrosio G., Oriente A., Napoli C., Palumbo G., Chiariello P., Marone G., Condorelli M., Triggiani M. (1994). Oxygen radicals inhibit human plasma acetylhydrolase, the enzyme that catabolizes platelet-activating factor. J. Clin. Investig..

[128] Howard A.D., Erickson K.L. (1996). Alteration of Macrophage responsiveness to platelet-activating factor by interferon-γ and lipopolysaccharide. Cell. Immunol..

[129] Huang Y.C., Kennedy T.P., Su Y.F., Watkins W.D., Whorton A.R., Piantadosi C.A. (1993). Protection against platelet-activating factor-induced injury by interferon inducer in perfused rabbit lung. J. Appl. Physiol..

[130] Perrone M.A., Gualtieri P., Gratteri S., Ali W., Sergi D., Muscoli S., Cammarano A., Bernardini S., Di Renzo L., Romeo F. (2019). Effects of postprandial hydroxytyrosol and derivates on oxidation of LDL, cardiometabolic state and gene expression. J. Cardiovasc. Med..

[131] Upadhyay S., Dixit M. (2015). Role of polyphenols and other phytochemicals on molecular signaling. Oxidative Med. Cell. Longev..

[132] Vázquez-Calvo Á., de Oya N., Martín-Acebes M.A., Garcia-Moruno E., Saiz J.-C. (2017). Antiviral properties of the natural polyphenols delphinidin and epigallocatechin gallate against the flaviviruses west nile virus, zika virus, and dengue virus. Front. Microbiol..

[133] Lin S.-C., Ho C.-T., Chuo W.-H., Li S., Wang T.T., Lin C.-C. (2017). Effective inhibition of MERS-CoV infection by resveratrol. BMC Infect. Dis..

[134] Utomo R.Y., Ikawati M., Meiyanto E. (2020). Revealing the Potency of Citrus and Galangal Constituents to Halt SARS-CoV-2 Infection. Preprints.

[135] Chen L., Hu C., Hood M., Zhang X., Zhang L., Kan J., Du J. (2020). A Novel combination of vitamin c, curcumin and glycyrrhizic acid potentially regulates immune and inflammatory response associated with coronavirus infections: A Perspective from system biology analysis. Nutrients.

[136] Theoharides T.C. (2020). COVID-19, pulmonary mast cells, cytokine storms, and beneficial actions of luteolin. Biofactors.

[137] Hensel A., Bauer R., Heinrich M., Spiegler V., Kayser O., Hempel G., Kraft K. (2020). Challenges at the Time of COVID-19: Opportunities and Innovations in antivirals from nature. Planta Med..

[138] Fragopoulou E., Nomikos T., Karantonis H.C., Apostolakis C., Pliakis E., Samiotaki M., Panayotou G., Antonopoulou S. (2007). Biological activity of acetylated phenolic compounds. J. Agric. Food Chem..

[139] Shah B.H., Nawaz Z., Pertani S.A., Roomi A., Mahmood H., Saeed S.A., Gilani A.H. (1999). Inhibitory effect of curcumin, a food spice from turmeric, on platelet-activating factor- and arachidonic acid-mediated platelet aggregation through inhibition of thromboxane formation and Ca2+ signaling. Biochem. Pharmacol..

[140] Keihanian F., Sahebkar A., Bagheri R.K., Johnston T.P., Sahebkar A. (2018). Curcumin, hemostasis, thrombosis, and coagulation. J. Cell. Physiol..

[141] Singh A., Shafi Z., Mahto S.K., Yadav S., Sankhwar R. (2020). Role and application of curcumin as an alternative therapeutic agent. Adv. Microb. Res..

[142] Tsoupras A.B., Fragopoulou E., Nomikos T., Iatrou C., Antonopoulou S., Demopoulos C.A. (2007). Characterization of the de novo biosynthetic enzyme of platelet activating factor, ddt-insensitive cholinephosphotransferase, of human mesangial cells. Mediat. Inflamm..

[143] Xanthopoulou M.N., Asimakopoulos D., Antonopoulou S., Demopoulos C.A., Fragopoulou E. (2014). Effect of robola and cabernet sauvignon extracts on platelet activating factor enzymes activity on U937 cells. Food Chem..

[144] Vlachogianni I.C., Fragopoulou E., Stamatakis G.M., Kostakis I.K., Antonopoulou S. (2015). Platelet activating factor (PAF) biosynthesis is inhibited by phenolic compounds in U-937 cells under inflammatory conditions. Prostaglandins Other Lipid Mediat..

[145] Yanoshita R., Chang H.W., Son K.H., Kudo I., Samejima Y. (1996). Inhibition of lyso PAF acetyltransferase activity by flavonoids. Inflamm. Res..

[146] Hartisch C., Kolodziej H., von Bruchhausen F. (1997). Dual Inhibitory activities of tannins from Hamamelis virginiana and related polyphenols on 5-Lipoxygenase and Lyso-PAF: Acetyl-CoA acetyltransferase1. Planta Med..

[147] Balestrieri M.L., Castaldo D., Balestrieri C., Quagliuolo L., Giovane A., Servillo L. (2003). Modulation by flavonoids of PAF and related phospholipids in endothelial cells during oxidative stress. J. Lipid Res..

[148] Nagumo S., Fukuju A., Takayama M., Nagai M., Yanoshita R., Samejima Y. (1999). Inhibition of LysoPAF Acetyltransferase activity by components of licorice root. Biol. Pharm. Bull..

[149] Shen T.Y. (1991). Chemical and biochemical characterization of lignan analogs as novel PAF receptor antagonists. Lipids.

[150] Guilleminault L., Williams E.J., Scott H.A., Berthon B.S., Jensen M., Wood L.G. (2017). Diet and asthma: Is it time to adapt our message?. Nutrients.

[151] Koloverou E., Panagiotakos D.B., Pitsavos C., Chrysohoou C., Georgousopoulou E.N., Grekas A., Christou A., Chatzigeorgiou M., Skoumas I.N., Tousoulis D. (2016). Adherence to Mediterranean diet and 10-year incidence (2002–2012) of diabetes: Correlations with inflammatory and oxidative stress biomarkers in the ATTICA cohort study. Diabetes Metab. Res. Rev..

[152] Panossian A., Brendler T. (2020). The role of adaptogens in prophylaxis and treatment of viral respiratory infections. Pharmaceuticals.

[153] Antonopoulou S., Fragopoulou E., Karantonis H.C., Mitsou E., Sitara M., Rementzis J., Mourelatos A., Ginis A., Phenekos C. (2006). Effect of Traditional greek mediterranean meals on platelet aggregation in normal subjects and in patients with type 2 diabetes mellitus. J. Med. Food.

[154] Karantonis H.C., Fragopoulou E., Antonopoulou S., Rementzis J., Phenekos C., Demopoulos C.A. (2006). Effect of fast-food Mediterranean-type diet on type 2 diabetics and healthy human subjects’ platelet aggregation. Diabetes Res. Clin. Pract..

[155] Maiorino M.I., Bellastella G., Longo M., Caruso P., Esposito K. (2020). Mediterranean Diet and COVID-19: Hypothesizing Potential benefits in people with diabetes. Front. Endocrinol..

[156] Lampropoulos C.E., Konsta M., Dradaki V., Roumpou A., Dri I., Papaioannou I. (2020). Effects of Mediterranean diet on hospital length of stay, medical expenses, and mortality in elderly, hospitalized patients: A 2-year observational study. Nutrition.

[157] Lo Buglio A., Bellanti F., Capurso C., Paglia A., Vendemiale G. (2019). Adherence to Mediterranean diet, malnutrition, length of stay and mortality in elderly patients hospitalized in internal medicine wards. Nutrients.

[158] Rizzo A., Sciorsci R.L., Magrone T., Jirillo E. (2020). Exploitation of some natural products for prevention and/or nutritional treatment of SARS-CoV2 infection. Endocr. Metab. Immune Disord. Drug Targets.

[159] Alkhatib A. (2020). Antiviral functional foods and exercise lifestyle prevention of coronavirus. Nutrients.

[160] Karantonis H.C., Antonopoulou S., Demopoulos C.A. (2002). Antithrombotic lipid minor constituents from vegetable oils. comparison between olive oils and others. J. Agric. Food Chem..

[161] Karantonis H.C., Tsantila N., Stamatakis G., Samiotaki M., Panayotou G., Antonopoulou S., Demopoulos C.A. (2008). Bioactive polar lipids in olive oil, pomace and waste byproducts. J. Food Biochem..

[162] Detopoulou M., Fragopoulou E., Mikellidi A., Vlachogianni Ι., Xanthopoulou Μ., Argyrou C., Nomikos T., Yannakoulia M., Antonopoulou S. (2020). Cardioprotective properties of a novel enriched yogurt with inhibitors of Platelet activating factor (PAF). Proc. Nutr. Soc..

[163] Panayiotou A., Samartzis D., Nomikos T., Fragopoulou E., Karantonis H.C., Demopoulos C.A., Zabetakis I. (2000). Lipid fractions with aggregatory and antiaggregatory activity toward platelets in fresh and fried cod (Gadus morhua): Correlation with platelet-activating factor and atherogenesis. J. Agric. Food Chem..

[164] Nasopoulou C., Nomikos T., Demopoulos C., Zabetakis I. (2007). Comparison of antiatherogenic properties of lipids obtained from wild and cultured sea bass (Dicentrarchus labrax) and gilthead sea bream (Sparus aurata). Food Chem..

[165] Nomikos T., Karantonis H.C., Skarvelis C., Demopoulos C.A., Zabetakis I. (2006). Antiatherogenic properties of lipid fractions of raw and fried fish. Food Chem..

[166] Rementzis J., Antonopoulou S., Demopoulos C.A. (1997). Identification and Study of gangliosides from *Scomber scombrus* muscle. J. Agric. Food Chem..

[167] Nasopoulou C., Tsoupras A.B., Karantonis H.C., Demopoulos C.A., Zabetakis I. (2011). Fish polar lipids retard atherosclerosis in rabbits by down-regulating PAF biosynthesis and up-regulating PAF catabolism. Lipids Health Dis..

[168] Nasopoulou C., Karantonis H.C., Andriotis M., Demopoulos C.A., Zabetakis I. (2008). Antibacterial and anti-PAF activity of lipid extracts from sea bass (Dicentrarchus labrax) and gilthead sea bream (Sparus aurata). Food Chem..

[169] Kafatos A., Verhagen H., Moschandreas J., Apostolaki I., Westerop J.J.M.V. (2000). Mediterranean Diet of Crete. J. Am. Diet. Assoc..

[170] Skiadas P., Lascaratos J. (2001). Dietetics in ancient Greek philosophy: Plato’s concepts of healthy diet. Eur. J. Clin. Nutr..

[171] Biluca F.C., da Silva B., Caon T., Mohr E.T.B., Vieira G.N., Gonzaga L.V., Vitali L., Micke G., Fett R., Dalmarco E.M. (2020). Investigation of phenolic compounds, antioxidant and anti-inflammatory activities in stingless bee honey (Meliponinae). Food Res. Int..

[172] Ranneh Y., Akim A.M., Ab Hamid H., Khaza’Ai H., Fadel A., Mahmoud A.M. (2019). Stingless bee honey protects against lipopolysaccharide induced-chronic subclinical systemic inflammation and oxidative stress by modulating Nrf2, NF-κB and p38 MAPK. Nutr. Metab..

[173] Pimentel R.B., Da Costa C.A., Albuquerque P.M., Junior S.D. (2013). Antimicrobial activity and rutin identification of honey produced by the stingless bee Melipona compressipes manaosensis and commercial honey. BMC Complement. Altern. Med..

[174] Mustafa M.Z., Shamsuddin S.H., Sulaiman S.A., Abdullah J.M. (2020). Anti-inflammatory properties of stingless bee honey may reduce the severity of pulmonary manifestations in COVID-19 Infections. Malays. J. Med Sci..

[175] Hossain K.S., Hossain M.G., Moni A., Rahman M.M., Rahman U.H., Alam M., Kundu S., Rahman M.M., Hannan M.A., Uddin M.J. (2020). Prospects of honey in fighting against COVID-19: Pharmacological insights and therapeutic promises. Heliyon.

[176] Hashem H.E. (2020). IN Silico approach of some selected honey constituents as sars-cov-2 main protease (COVID-19) inhibitors. Eurasian J. Med. Oncol..

[177] Ahmed A., Khan R.A., Azim M.K., Saeed S.A., Mesaik M.A., Ahmed S., Imran I. (2011). Effect of natural honey on human platelets and blood coagulation proteins. Pak. J. Pharm. Sci..

[178] Koussissis G., Semidalas E., Hadjistavrou E., Kalyvas V., Antonopoulou S., Demopoulos C.A. (1994). PAF antagonists in food: Isolation and identification of PAF antagonists in honey and wax. Rev. Fr. Corps Gras.

[179] Mardani R., Alamdary A., Nasab S.M., Gholami A., Ahmadi N. (2020). Association of vitamin D with the modulation of the disease severity in COVID-19. Virus Res..

[180] Lau F.H., Majumder R., Torabi R., Saeg F., Hoffman R., Cirillo J.D., Greiffenstein P. (2020). Vitamin D Insufficiency is prevalent in severe Covid-19. MedRxiv.

[181] Panagiotou G., Tee S.A., Ihsan Y., Athar W., Marchitelli G., Kelly D., Boot C.S., Stock N., Macfarlane J., Martineau A.R. (2020). Low serum 25-hydroxyvitamin D (25[OH]D) levels in patients hospitalized with COVID-19 are associated with greater disease severity. Clin. Endocrinol..

[182] Antonopoulou S., Semidalas C.E., Koussissis S., Demopoulos C.A. (1996). Platelet-Activating factor (PAF) Antagonists in foods: A study of lipids with paf or anti-paf-like activity in cow’s milk and yogurt. J. Agric. Food Chem..

[183] Lordan R., Vidal N.P., Pham T.H., Tsoupras A., Thomas R.H., Zabetakis I. (2020). Yoghurt fermentation alters the composition and antiplatelet properties of milk polar lipids. Food Chem..

[184] Lordan R., Walsh A.M., Crispie F., Finnegan L., Cotter P.D., Zabetakis I. (2019). The effect of ovine milk fermentation on the antithrombotic properties of polar lipids. J. Funct. Foods.

[185] Megalemou K., Sioriki E., Lordan R., Dermiki M., Nasopoulou C., Zabetakis I. (2017). Evaluation of sensory and in vitro anti-thrombotic properties of traditional Greek yogurts derived from different types of milk. Heliyon.

[186] Shaghaghi N. (2020). Molecular Docking Study of Novel COVID-19 Protease with Low Risk Terpenoides Compounds of Plants. Chemrixiv.

[187] Wang K.-L., Li Z.-Q., Cao Z.-Y., Ke Z.-P., Cao L., Wang Z.-Z., Xiao W. (2017). Effects of ginkgolide A, B and K on platelet aggregation. Zhongguo Zhong Yao Za Zhi.

[188] Detopoulou P., Aggeli M., Andrioti E., Detopoulou M. (2016). Macronutrient content and food exchanges for 48 Greek Mediterranean dishes. Nutr. Diet..

[189] Phillips C., Poyser Norman L. (1978). Inhibition of platelet aggregation by onion extracts. Lancet.

[190] Lim H., Kubota K., Kobayashi A., Seki T., Ariga T. (1999). Inhibitory effect of sulfur-containing compounds in *Scorodocarpus borneensis* Becc. on the Aggregation of rabbit platelets. Biosci. Biotechnol. Biochem..

[191] Fragopoulou E., Detopoulou P., Nomikos T., Pliakis E., Panagiotakos D., Antonopoulou S. (2012). Mediterranean wild plants reduce postprandial platelet aggregation in patients with metabolic syndrome. Metabolism.

[192] Wong W.-T., Ismail M., Imam M.U., Zhang Y.-D. (2016). Modulation of platelet functions by crude rice (Oryza sativa) bran policosanol extract. BMC Complement. Altern. Med..

[193] Rungratanawanich W., Cenini G., Mastinu A., Sylvester M., Wilkening A., Abate G., Bonini S., Aria F., Marziano M., Maccarinelli G. (2019). γ-Oryzanol improves cognitive function and modulates hippocampal proteome in mice. Nutrients.

[194] Bousquest J., Cristol J.-P., Czarlewski W., Anto J.M., Martineau A., Haahtela T., Fonseca S.C., Iaccarino G., Blain H., Fiocchi A. (2020). Nrf2-interacting nutrients and COVID-19: Time for research to develop adaptation strategies. Clin. Transl. Allergy.

[195] Tufekci K.U., Bayin E.C., Genc S., Genc K. (2011). The Nrf2/ARE Pathway: A Promising target to counteract mitochondrial dysfunction in parkinson’s disease. Park. Dis..

[196] Fragopoulou E., Antonopoulou S. (2020). The French paradox three decades later: Role of inflammation and thrombosis. Clin. Chim. Acta.

[197] Xanthopoulou M.N., Kalathara K., Melachroinou S., Arampatzi-Menenakou K., Antonopoulou S., Yannakoulia M., Fragopoulou E. (2016). Wine consumption reduced postprandial platelet sensitivity against platelet activating factor in healthy men. Eur. J. Nutr..

[198] Argyrou C., Vlachogianni I., Stamatakis G., Demopoulos C.A., Antonopoulou S., Fragopoulou E. (2017). Postprandial effects of wine consumption on platelet activating factor metabolic enzymes. Prostaglandins Other Lipid Mediat..

[199] Fragopoulou E., Antonopoulou S., Demopoulos C.A. (2002). Biologically active lipids with antiatherogenic properties from white wine and must. J. Agric. Food Chem..

[200] Fragopoulou E., Nomikos T., Antonopoulou S., Mitsopoulou C.A., Demopoulos C.A. (2000). Separation of biologically active lipids from red wine. J. Agric. Food Chem..

[201] Fragopoulou E., Nomikos T., Tsantila N., Mitropoulou A., Zabetakis I., Demopoulos C.A. (2001). Biological activity of total lipids from red and white wine/must. J. Agric. Food Chem..

[202] Fragopoulou E., Antonopoulou S., Nomikos T., Demopoulos C.A. (2003). Structure elucidation of phenolic compounds from red/white wine with antiatherogenic properties. Biochim. Biophys. Acta Mol. Cell Biol. Lipids.

[203] Choleva M., Boulougouri V., Panara A., Panagopoulou E., Chiou A., Τhomaidis Ν.S., Antonopoulou S., Fragopoulou E. (2019). Evaluation of anti-platelet activity of grape pomace extracts. Food Funct..

[204] Mori T.A., Beilin L.J., Burke V., Morris J., Ritchie J. (1997). Interactions between dietary fat, fish, and fish oils and their effects on platelet function in men at risk of cardiovascular disease. Arter. Thromb. Vasc. Biol..

[205] Grimminger F., Mayser P., Papavassilis C., Thomas M., Schlotzer E., Heuer K.-U., Führer D., Hinsch K.-D., Walmrath D., Schill W.-B. (1993). A double-blind, randomized, placebo-controlled trial of n-3 fatty acid based lipid infusion in acute, extended guttate psoriasis. J. Mol. Med..

[206] Gavriil L., Detopoulou M., Petsini F., Antonopoulou S., Fragopoulou E. (2019). Consumption of plant extract supplement reduces platelet activating factor-induced platelet aggregation and increases platelet activating factor catabolism: A randomised, double-blind and placebo-controlled trial. Br. J. Nutr..

[207] Chung K.F., Dent G., McCusker M., Guinot P., Page C., Barnes P.J. (1987). Effect of a ginkgolide mixture (bn 52063) in antagonising skin and platelet responses to platelet activating factor in man. Lancet.

[208] Vijayaraghavan K., Szerlip H.M., Ballantyne C.M., Bays H.E., Philip S., Doyle R.T., Juliano R.A., Granowitz C. (2019). Icosapent ethyl reduces atherogenic markers in high-risk statin-treated patients with stage 3 chronic kidney disease and high triglycerides. Postgrad. Med..

[209] Brinton E.A., Ballantyne C.M., Bays H.E., Kastelein J.J.P., Braeckman R.A., Soni P.N. (2013). Effects of icosapent ethyl on lipid and inflammatory parameters in patients with diabetes mellitus-2, residual elevated triglycerides (200–500 mg/dL), and on statin therapy at LDL-C goal: The ANCHOR study. Cardiovasc. Diabetol..

[210] Woodman R.J., Mori T.A., Burke V., Puddey I.B., Barden A., Watts G.F., Beilin L.J. (2003). Effects of purified eicosapentaenoic acid and docosahexaenoic acid on platelet, fibrinolytic and vascular function in hypertensive type 2 diabetic patients. Atheroscler..

[211] Kastelein J.J.P., Maki K.C., Susekov A., Ezhov M., Nordestgaard B.G., Machielse B.N., Kling D., Davidson M.H. (2014). Omega-3 free fatty acids for the treatment of severe hypertriglyceridemia: The Epanova for lowering very high triglycerides (EVOLVE) trial. J. Clin. Lipidol..

[212] Mosca L., Ballantyne C.M., Bays H.E., Guyton J.R., Philip S., Doyle R.T., Juliano R.A. (2017). Usefulness of icosapent ethyl (eicosapentaenoic acid ethyl ester) in women to lower triglyceride levels (results from the marine and anchor trials). Am. J. Cardiol..

[213] Krantz M.J., Havranek E.P., Pereira R.I., Beaty B., Mehler P.S., Long C.S. (2015). Effects of omega-3 fatty acids on arterial stiffness in patients with hypertension: A randomized pilot study. J. Negat. Results Biomed..

[214] Wooten J.S., Nambi P., Gillard B.K., Pownall H.J., Coraza I., Scott L.W., Nambi V., Ballantyne C.M., Balasubramanyam A. (2013). intensive lifestyle modification reduces Lp-PLA2 in dyslipidemic HIV/HAART patients. Med. Sci. Sports Exerc..

[215] Kim M., Jeung S.R., Jeong T.-S., Lee S.-H., Lee J.H. (2014). Replacing with whole grains and legumes reduces Lp-PLA2 activities in plasma and PBMCs in patients with prediabetes or T2D. J. Lipid Res..

[216] Morris J., Burke V., Mori T.A., Vandongen R., Beilin L.J. (1995). Effects of garlic extract on platelet aggregation: A randomized placebo-controlled double-blind study. Clin. Exp. Pharmacol. Physiol..

[217] Asztalos I.B., Gleason J.A., Sever S., Gedik R., Asztalos B.F., Horvath K.V., Dansinger M.L., Lamon-Fava S., Schaefer E.J. (2016). Effects of eicosapentaenoic acid and docosahexaenoic acid on cardiovascular disease risk factors: A randomized clinical trial. Metabolism.

[218] Maki K.C., Bays H.E., Dicklin M.R., Johnson S.L., Shabbout M. (2011). Effects of prescription omega-3-acid ethyl esters, coadministered with atorvastatin, on circulating levels of lipoprotein particles, apolipoprotein CIII, and lipoprotein-associated phospholipase A2 mass in men and women with mixed dyslipidemia. J. Clin. Lipidol..

[219] Bays H.E., Ballantyne C.M., Kastelein J.J., Isaacsohn J.L., Braeckman R.A., Soni P.N. (2011). Eicosapentaenoic acid ethyl ester (amr101) therapy in patients with very high triglyceride levels (from the multi-center, placebo-controlled, randomized, double-blind, 12-week study with an open-label extension [marine] trial). Am. J. Cardiol..

[220] Dunbar R.L., Nicholls S.J., Maki K.C., Roth E.M., Orloff D.G., Curcio D., Johnson J., Kling D., Davidson M.H. (2015). Effects of omega-3 carboxylic acids on lipoprotein particles and other cardiovascular risk markers in high-risk statin-treated patients with residual hypertriglyceridemia: A randomized, controlled, double-blind trial. Lipids Health Dis..

[221] Hedengran A., Szecsi P.B., Dyerberg J., Harris W.S., Stender S. (2014). N-3 PUFA Esterified to glycerol or as ethyl esters reduce non-fasting plasma triacylglycerol in subjects with hypertriglyceridemia: A randomized trial. Lipids.

[222] Mayer K., Fegbeutel C., Hattar K., Sibelius U., Krämer H.-J., Heuer K.-U., Temmesfeld-Wollbrück B., Gokorsch S., Grimminger F., Seeger W. (2003). ω-3 vs. ω-6 lipid emulsions exert differential influence on neutrophils in septic shock patients: Impact on plasma fatty acids and lipid mediator generation. Intensive Care Med..

[223] Kerely C.P., Hutchinson K., Bramham J., McGowan A., Faul J., Cormican L. (2017). Vitamin D Improves selected metabolic parameters but not neuropsychological or quality of life indices in osa: A Pilot Study. J. Clin. Sleep Med..

[224] Mori T.A., Vandongen R., Mahanian F., Douglas A. (1992). Plasma lipid levels and platelet and neutrophil function in patients with vascular disease following fish oil and olive oil supplementation. Metabolism.

[225] Ballantyne C.M., Bays H.E., Kastelein J.J., Stein E., Isaacsohn J.L., Braeckman R.A., Soni P.N. (2012). Efficacy and safety of eicosapentaenoic acid ethyl ester (amr101) therapy in statin-treated patients with persistent high triglycerides (from the anchor study). Am. J. Cardiol..

[226] Miller M., Ballantyne C.M., Bays H.E., Granowitz C., Doyle R.T., Juliano R.A., Philip S. (2019). Effects of icosapent ethyl (eicosapentaenoic acid ethyl ester) on atherogenic lipid/lipoprotein, apolipoprotein, and inflammatory parameters in patients with elevated high-sensitivity c-reactive protein (from the anchor study). Am. J. Cardiol..

[227] Beulens J.W.J., van den Berg R., Kok F.J., Helander A., Vermunt S.H.F., Hendriks H.F. (2008). Moderate alcohol consumption and lipoprotein-associated phospholipase A2 activity. Nutr. Metab. Cardiovasc. Dis..

[228] García-Conesa M.-T., Philippou E., Pafilas C., Massaro M., Quarta S., Andrade V., Jorge R., Chervenkov M., Ivanova T., Dimitrova D. (2020). Exploring the Validity of the 14-Item Mediterranean Diet Adherence Screener (MEDAS): A Cross-National Study in Seven European Countries around the Mediterranean Region. Nutrients.

[229] Pelucchi C., Galeone C., Negri E., La Vecchia C. (2010). Trends in adherence to the Mediterranean diet in an Italian population between 1991 and 2006. Eur. J. Clin. Nutr..

[230] Di Renzo L., Gualtieri P., Pivari F., Soldati L., Attinà A., Cinelli G., Leggeri C., Caparello G., Barrea L., Scerbo F. (2020). Eating habits and lifestyle changes during COVID-19 lockdown: An Italian survey. J. Transl. Med..

[231] Ruiz-Roso M.B., de Carvalho Padilha P., Mantilla-Escalante D.C., Ulloa N., Brun P., Acevedo-Correa D., Arrantes Ferreira Peres W., Martorell M., Aires M.T., de Oliveira Cardoso L. (2020). Covid-19 Confinement and Changes of Adolescent’s Dietary Trends in Italy, Spain, Chile, Colombia and Brazil. Nutrients.

[232] Rodríguez-Pérez C., Molina-Montes E., Verardo V., Artacho R., García-Villanova B., Guerra-Hernández E.J., Ruiz-López M.-D. (2020). Changes in dietary behaviours during the COVID-19 Outbreak Confinement in the Spanish COVIDiet Study. Nutrients.

[233] PRESS RELEASE—The Role of Nutrition in the COVID-19 Era. https://www.hda.gr/deltio-typoy-o-rolos-tis-diatrofis-stin-epochi-tis-covid-19-prolipsi-antimetopisi-kai-o-paragontas-tis-pachusarkias/.

